# Global patterns of Middle East respiratory syndrome coronavirus (MERS-CoV) prevalence and seroprevalence in camels: A systematic review and meta-analysis

**DOI:** 10.1016/j.onehlt.2023.100561

**Published:** 2023-05-08

**Authors:** Md. Mazharul Islam, Hamida Khanom, Elmoubashar Farag, Zarin Tasnim Mim, Pragalathan Naidoo, Zilungile Lynette Mkhize-Kwitshana, Markos Tibbo, Ariful Islam, Ricardo J. Soares Magalhaes, Mohammad Mahmudul Hassan

**Affiliations:** a Department of Animal Resources, Ministry of Municipality, Doha, Qatar; b Faculty of Veterinary Medicine, Chattogram Veterinary and Animal Sciences University, Chattogram 4225, Bangladesh; c Ministry of Public Health, Doha, Qatar; d Discipline of Medical Microbiology, College of Health Sciences, University of KwaZulu Natal, Durban 4000, South Africa; e Division of Research Capacity Development, Medical Research Council, Tygerberg, Cape Town 7505, South Africa; f Food and Agriculture Organization of the United Nations (FAO), Subregional Office for the Gulf Cooperation Council States and Yemen, Abu Dhabi, United Arab Emirates; g EcoHealth Alliance, NY 10001, USA; h Queensland Alliance for One Health Sciences, School of Veterinary Science, The University of Queensland, QLD 4343, Australia; i Children Health and Environment Program, UQ Child Health Research Centre, The University of Queensland, QLD 4343, Australia

**Keywords:** MERS-CoV, Risk factors, Prevalence, Seroprevalence, Camel, Global pattern

## Abstract

The Middle East respiratory syndrome Coronavirus (MERS-CoV) is one of the human coronaviruses that causes severe respiratory infection. Bats are considered to be the natural reservoir, where dromedary camels (DC) are the intermediate hosts of the virus. The current study was undertaken to provide an update on global distribution of the virus in camels, and to investigate the pooled prevalence and camel-associated risk factors of infection. After registration of the review protocol in the Open Science Framework, data searches were conducted on 18 April 2023 through Embase, PubMed, Scopus, and Web of Science. Considering only natural MERS-CoV infection in camels, 94 articles were selected for data curation through blind screening by two authors. Meta-analysis was conducted to estimate the pooled prevalence and to evaluate camel-associated risk factors. Finally, the results were presented in forest plots. The reviewed articles tested 34 countries, of which camels of 24 countries were seropositive and in 15 countries they were positive by molecular method. Viral RNA was detected in DC. Non-DC, such as bactrian camels, alpaca, llama, and hybrid camels were only seropositive. The global estimated pooled seroprevalence and viral RNA prevalence in DC were 77.53% and 23.63%, respectively, with the highest prevalence in West Asia (86.04% and 32.37% respectively). In addition, 41.08% of non-DC were seropositive. The estimated pooled prevalence of MERS-CoV RNA significantly varied by sample types with the highest in oral (45.01%) and lowest in rectal (8.42%) samples; the estimated pooled prevalence in nasal (23.10%) and milk (21.21%) samples were comparable. The estimated pooled seroprevalence in <2 years, 2–5 years, and > 5 years age groups were 56.32%, 75.31%, and 86.31%, respectively, while viral RNA prevalence was 33.40%, 15.87%, and 13.74%, respectively. Seroprevalence and viral RNA prevalence were generally higher in females (75.28% and 19.70%, respectively) than in males (69.53% and 18.99%, respectively). Local camels had lower estimated pooled seroprevalence (63.34%) and viral RNA prevalence (17.78%) than those of imported camels (89.17% and 29.41%, respectively). The estimated pooled seroprevalence was higher in camels of free-herds (71.70%) than confined herds (47.77%). Furthermore, estimated pooled seroprevalence was higher in samples from livestock markets, followed by abattoirs, quarantine, and farms but viral RNA prevalence was the highest in samples from abattoirs, followed by livestock markets, quarantine, and farms. Risk factors, such as sample type, young age, female sex, imported camels, and camel management must be considered to control and prevent the spread and emergence of MERS-CoV.

## 1 Background

The Middle East respiratory syndrome Coronavirus (MERS-CoV) is one of the seven known human coronaviruses (HCoV) and belongs to the β-CoV lineage C [1]. Four HCoV, namely HCoV-OC43, HCoV-229E, HCoV-NL63, and HCoV-HKU1, cause mild respiratory tract infection, whereas three HCoV, that include SARS-CoV, MERS-CoV, and SARS-CoV-2 cause severe respiratory infection [2,3]. Middle East Respiratory Syndrome (MERS) is caused by MERS-CoV, first reported in 2012 in Saudi Arabia [4,5]. In humans, MERS-CoV causes asymptomatic infection to rapidly progressive acute respiratory distress, which may eventually lead to septic shock, multiorgan failure, and death [6]. Among the HCoVs, MERS-CoV has the highest case fatality rate in humans (>35%). Between 2012 and 17 October 2022, a total of 2600 laboratory-confirmed human MERS cases were documented worldwide, with 935 deaths [7]. Although the virus has been reported in humans from at least 27 countries worldwide, most of the cases were seen in the Middle East. Most of the index cases in the Middle East region are directly or indirectly associated with DC, one of the most highly valued livestock animals in this region [7]. The index cases from outside the Middle East region were thought to have contracted the infection from Middle East [8,9].

Although the disease first emerged in 2012, the pathogen link with DC was only discovered in 2014 [10]. The origin of the virus is still unknown. Evidence indicates that bats are the host of the ancestor MERS-CoV; they are no longer seem to be implicated in contemporaneous MERS-CoV epidemiology. DC are considered secondary or intermediate reservoirs [11,12]. The virus infects humans sporadically, and humans are considered to be the pathogen's evolutionally terminal host [13]. Notably, camels infected with MERS-CoV show no symptoms or occasionally mild lesions in the upper respiratory tract. Non-dromedary camels (non-DC), such as bactrian camels (BC) and new world camels (NWC), including llamas, alpacas, and guanacos were found to be seropositive [14]. To reduce the human prevalence of MERS, it is essential to understand the prevalence of the virus in camels and natural host associated risk factors. A previous systematic review showed that DC in 20 countries were seropositive against MERS-CoV, and 13 countries were positive for viral RNA, with a global seroprevalence of 70% [14]. However, there is no updated pooled estimate of the viral prevalence among camels in the context of host-related risk factors, which would be important to inform risk assessments for DC management practices. Therefore, the current study was undertaken to update the global distribution of MERS-CoV, and to investigate the pooled prevalence and the associated risk factors of MERS-CoV in camels.

## 2 Methods

**Fig. 1:**
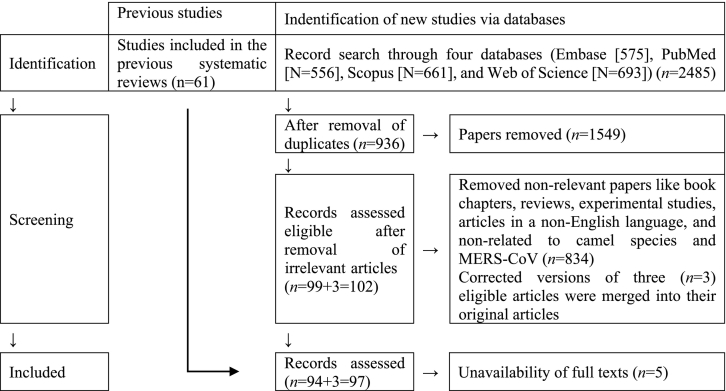
Systematic review PRISMA 2020 flow diagram describing the selection of published articles on MERS-CoV in camels and the inclusion/exclusion process used in the study.

The systematic review protocol followed the Preferred Reporting Items for Systematic Reviews and Meta-Analysis 2020 (PRISMA 2020) guidelines [[15], [16], [17]] (Fig. 1 and Supplementary file 1), which was registered in Open Science Framework [18]. One author performed electronic data searches and initial duplicate removal. Two authors blindly examined the eligibility of the initially screened articles with a set of predetermined selection criteria and extracted data from the eligible articles. After that, two authors evaluated the data and compiled into a single document. Finally, two authors conducted the data analysis.

### 2.1 Systematic data search

In the beginning, we studied the three previously published systematic reviews on MERS-CoV in camels [14,19,20] and listed the reviewed articles. Then systematic data searches were conducted on Embase, PubMed, Scopus, and Web of Science on 18 April 2023 using the keywords: ((Camel OR Dromedary OR Bactrian OR Alpaca OR Llama OR Guanaco OR "Hybrid came") AND (“Middle East respiratory syndrome” OR MERS OR “Middle East respiratory syndrome coronavirus” OR MERS-CoV)), without any timeframe of publication. Following a similar approach to a previous systematic review [21], literature searches were filtered by selecting Title/Abstract in PubMed, TITLE-ABS-KEY in Scopus, and Topic in Web of Science. The search results were transferred to EndNote X9 (Clarivate Analytics, Philadelphia, PA, USA) and duplicates were identified and removed. After that, screened citations were transferred to the Rayyan system (https://rayyan.qcri.org/) to filter articles with the inclusion criteria: (1) camelid: all types of camels including dromedary, bactrian, llama, alpaca, guanaco, and hybrid camels; (2) natural infection of MERS-CoV; (3) case study, outbreak, prevalence, and risk factor studies; and exclusion criteria (1) experimental study, (2) Non-MERS-CoV, (3) articles in a non-English language, review, and conference proceedings. After selecting the eligible studies, the full texts of the articles were collected through Endnote, PubMed, Science Direct, and ResearchGate. Articles that were unavailable through these databases were collected upon request from the Qatar National Library document delivery system (https://qnl.qa/en). The PRISMA-S statement of the literature searches, abstract and final reporting in the current study has been presented in Supplementary file 2.

### 2.2 Quality assessment of the selected studies

Two authors blindly conducted the risk of bias assessment of the included studies. Afterwards, one author compiled the evaluation results into a single document (Microsoft Excel spreadsheet). The risk of bias assessment was conducted using ten questions with the answer of “yes”, “no”, “unclear”, and “not applicable” following the modified version of the critical appraisal tool for prevalence studies reported previously [22,23]. A score was calculated as the percentage of yes on total yes, no, and unclear answers for each study. The studies were categorized into three groups based on their score: low (≤40%), intermediate (>40% to <70%), and high (≥70%).

### 2.3 Data extraction

Based on the availability of data in the included articles, the extracted variables included country of study, global region, year of sampling, camel-specific data (species, gender, and age), sample type (nasal swab, rectal swab, milk, and serum), source of the camels (local and imported), camel management system (free herd and confined herd), sampling site (farm, abattoir, market, and quarantine) and other factors (if any article specified, such as season) (Supplementary file 3). The regions of the world included South Asia, East Asia, West Asia, North Africa, West Africa, East Africa, and other countries. The age of camels was classified as <2 years, 2–5 years, and > 5 years. Camels were classified as belonging to free herd when studies reported from nomadic, pastoralist, or frequent contact with outside camels for travel, grazing, drafting, or other reasons. Confined herd was considered when camels a herd were kept through general farming system, research facility, or conservation center and had little or no contact with outside camels.

### 2.4 Data analysis

The aggregated data was transcribed into a Microsoft Excel (MS Office, 2019) spreadsheet. A descriptive statistic of the included articles was conducted using Rstudio. MERS-CoV global distribution map was prepared using ArcMap 10.8. The temporal patterns of the prevalence and seroprevalence were expressed in graph based on the sample collection year by the studied researches. A quantitative meta-analysis was performed using STATA/IC-13.0 (Stata Corp, 4905 Lakeway Drive, College Station, Texas 77,845, USA). Only DC had enough data to identify host-associated risk variables; hence only DC data were included for meta-analysis for the risk factor identification. The meta-analyses results were illustrated in forest plots. In addition, funnel plots were generated to assess the possibility of publication bias. All studies reporting zero prevalence of MERS-CoV in camels were omitted as such data could not be used in STATA for inclusion in the meta-analysis.

## 3 Results

### 3.1 Descriptive statistics

The literature search and screening yielded 99 eligible articles (Fig. 1) published between 2013 and 2023. However, five articles were excluded as the full texts were not available. Among the 94 included articles, 13 (13.98%, 95%CI: 7.94–23.08) used archived samples (between the years of 1983 and 2010), while 90 (95.74%, 95%CI: 89.56–98.33) used samples that were collected after the first outbreak report of human MERS in 2012 (Table 1). A total of 33 (35.10%, 95%CI: 26.22–45.67) articles focused only on screening of antibodies against the virus, and 25 (26.60%, 95%CI: 18.71–36.32) articles tested for the presence of viral RNA. Ninety-one (96.81%, 95%CI: 91.10–98.91), 10 (10.64%, 95%CI: 5.88–18.49), and 3 (3.19%, 95%CI: 1.09–8.97) articles investigated DC, BC, and NWC, respectively. The majority of the studies were from West Asia (*n* = 17, 18.08%, 95%CI: 11.61–27.07), followed by East Africa (*n* = 13, 13.83%, 95%CI: 8.26–22.24), North Africa (*n* = 12, 12.76%, 95%CI: 7.46–21.00), and West Africa (*n* = 9, 9.00%, 95%CI: 5.12–17.20). Seventy-six (80.85%, 95%%CI: 71.75–87.53) articles were characterized as high-quality articles with an average quality score of 92 out of 100, whereas the rest 18 (19.15%, 95%CI: 12.47–28.25) articles were of intermediate quality, with an average score of 66 out of 100. The funnel plots (Supplementary file 4) showed asymmetry, indicating publication bias for seroprevalence and viral RNA prevalence as many of the points fell outside the funnels. As no low-quality article was in the included studies, all were used in the meta-analysis.

**Table 1:** Characteristics of the reviewed articles.

Characteristics	Number of Articles (%, 95%CI)
Publication year	
2013–2015	33 (35.11, 26.22–45.17)
2016–2018	32 (34.04, 25.26–44.08)
2019–2021	23 (24.47, 16.89–34.04)
2022–2023	6 (6.38, 2.96–13.23)

Sampling time	
Before 2013 (Archived sample)	13 (13.83, 8.26–22.24)
2013 and after	90 (95.74, 89.56–98.33)

MERS-CoV test	
Antibody detection test only	33 (35.10 26.22–45.67)
Detection of viral RNA only	25 (26.60, 18.71–36.32)
Both antibodies and viral RNA detection	36 (38.29, 29.44–48.40)

Camel type	
Dromedary camel	91 (96.81, 91.10–98.91)
Bactrian camel	10 (10.64, 5.88–18.91)
New world camel (Llama, Alpaca, Guanaco, Hybrid Camel)	3 (3.19, 1.09–8.97)

Study region	
*Asia*	
East Asia (China, Japan, Mongolia)	6 (6.38, 2.96–13.24)
South Asia (Bangladesh, Pakistan)	3 (3.19, 1.09–8.97)
Western Asia (Iraq, Israel, Jordan, Oman, Qatar, Saudi Arabia, UAE)	17 (18.08, 11.61–27.07)
*Africa*	
North Africa (Egypt, Morocco, Tunisia)	12 (12.76, 7.46–21.00)
East Africa (Ethiopia, Kenya, Somalia, Sudan, Uganda)	13 (13.83, 8.26–22.24)
West Africa (Burkina faso, Canary Island, Mali, Nigeria, Senegal)	9 (9.57, 5.12–17.20)
*Other parts of the world*	
Australia	2 (2.13, 0.59–7.43)
Central Asia (Kazakhstan)	3 (3.19, 1.09–8.97)
Europe (Germany, Netherlands)	2 (2.13, 0.59–7.43)
North America (USA, Canada)	1 (1.06, 0.01–5.78)
South America (Cheli)	1 (1.06, 0.01–5.78)

### 3.2 Global distribution of MERS-CoV

**Fig. 2:**
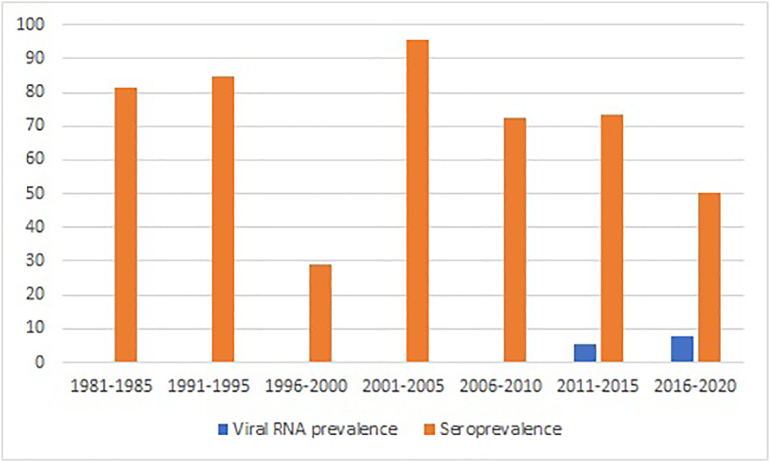
Temporal trends of MERS-CoV RNA and seroprevalence worldwide.

**Fig. 3:**
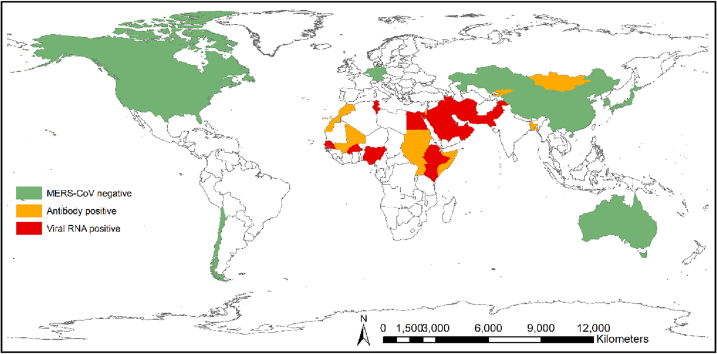
Global distribution of MERS-CoV in camels.

The articles studied MERS-CoV in 34 countries/territories in different regions of the world either by immunologic, molecular, or both methods. The temporal prevalence of both viral RNA and seroprevalence globally among dromedary camels has been shown in Fig. 2. Antibodies against MERS-CoV were tested in 33 countries, where 24 were seropositive (Fig. 3). Viral RNA was tested in 22 countries, of which positive camels were detected in 15 countries. Several countries in Asia (Japan and South Korea), Europe (the Netherlands and Germany), America (the United States and Canada), and Australia tested MERS-CoV either by immunologic or molecular methods and got negative results. Notably, the viral RNA was detected only in DC. Non-DC, such as BC (Kazakhstan, Mongolia and UAE), alpaca (Israel and Qatar), llama (Israel), and hybrid camels (UAE), were only seropositive.

### 3.3 MERS-CoV prevalence and seroprevalence

**Fig. 4:**
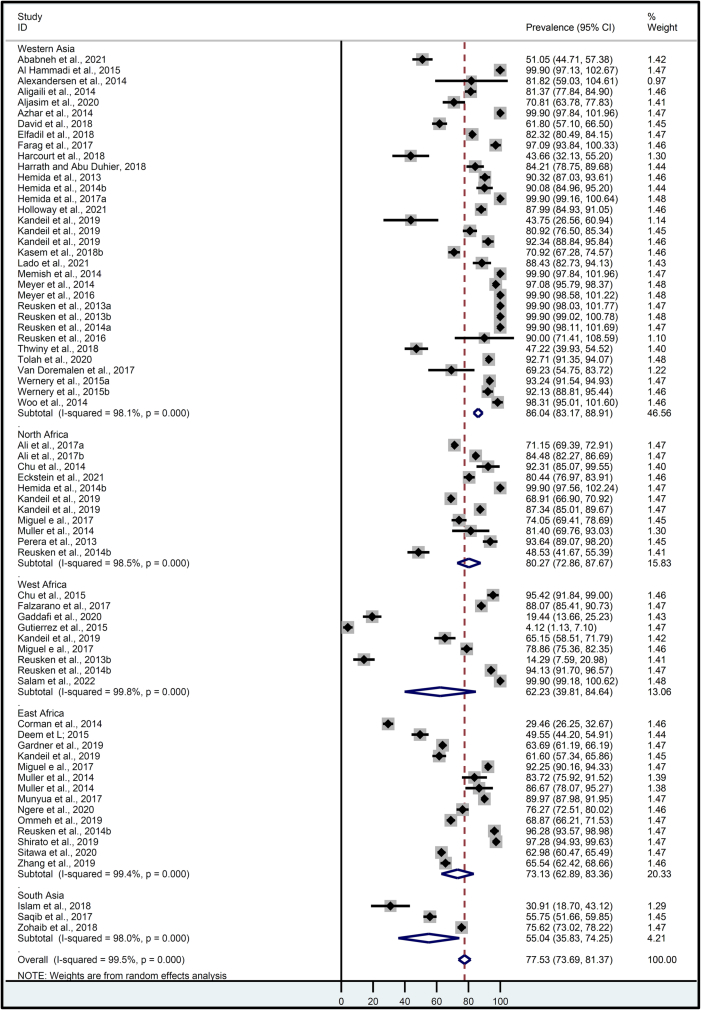
Forest plot showing the pooled global seroprevalence of MERS-CoV in dromedary camels. The central black square represents point estimates, whereas the square size represents the weight of each study in the meta-analysis.

**Fig. 5:**
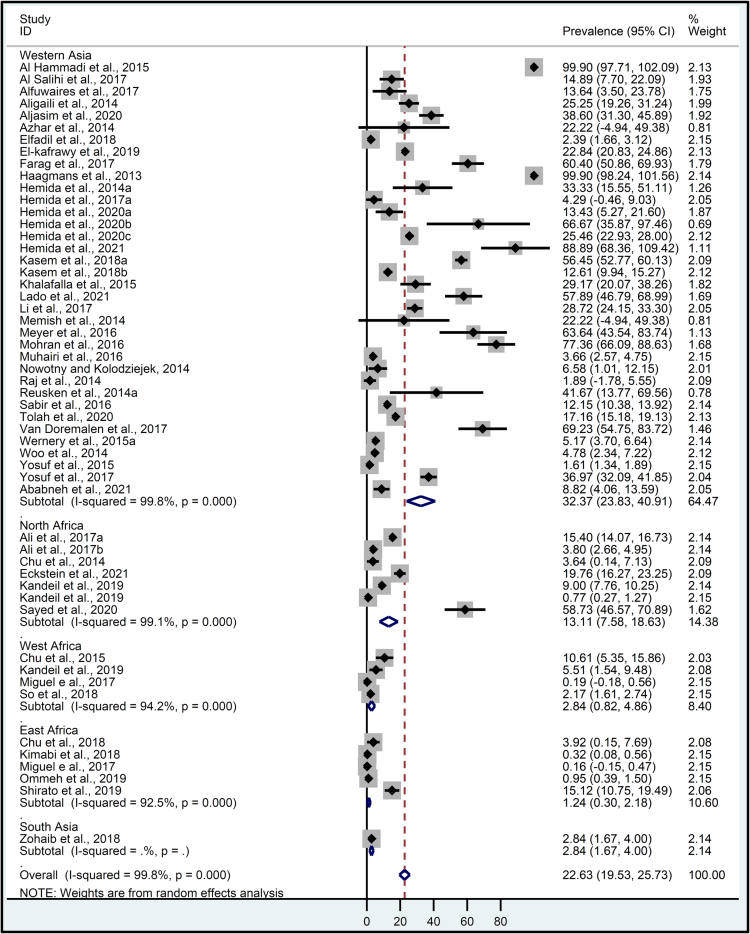
Forest plot showing the pooled global prevalence of MERS-CoV RNA in dromedary camels. The central black square represents point estimates, whereas the square size represents the weight of each study in the meta-analysis.

**Fig. 6:**
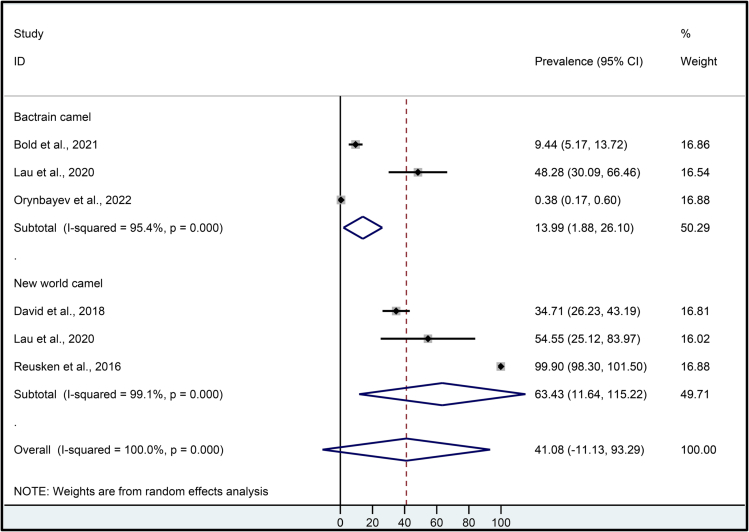
Forest plot showing the pooled seroprevalence of MERS-CoV in non-dromedary camels. The central black square represents point estimates, whereas the square size represents the weight of each study in the meta-analysis.

The global seroprevalence of MERS-CoV in DC was 77.53% (95%CI: 73.69–81.37) (Fig. 4). DC in the Western Asia had the highest seroprevalence (86.04%, 95%CI: 83.17–88.91), while the lowest seroprevalent region was South Asia (55.04%, 95%CI: 35.83–74.25). In Africa, North Africa was the highest seroprevalent region (80.27%, 95%CI: 72.86–87.67). Around 23.63% of DC were positive for viral RNA globally. The prevalence of viral RNA was highest in West Asia (32.37%, 95%CI: 23.83–40.91) and lowest in East Africa (1.24%, 95%CI: 0.30–2.18) (Fig. 5). In addition, 41.08% (95%CI: −11.13-93.29) of the non-DC were seropositive for MERS-CoV (Fig. 6).

### 3.4 Risk factors associated with MERS-CoV prevalence and seroprevalence

#### 3.4.1 Sample type

**Fig. 7:**
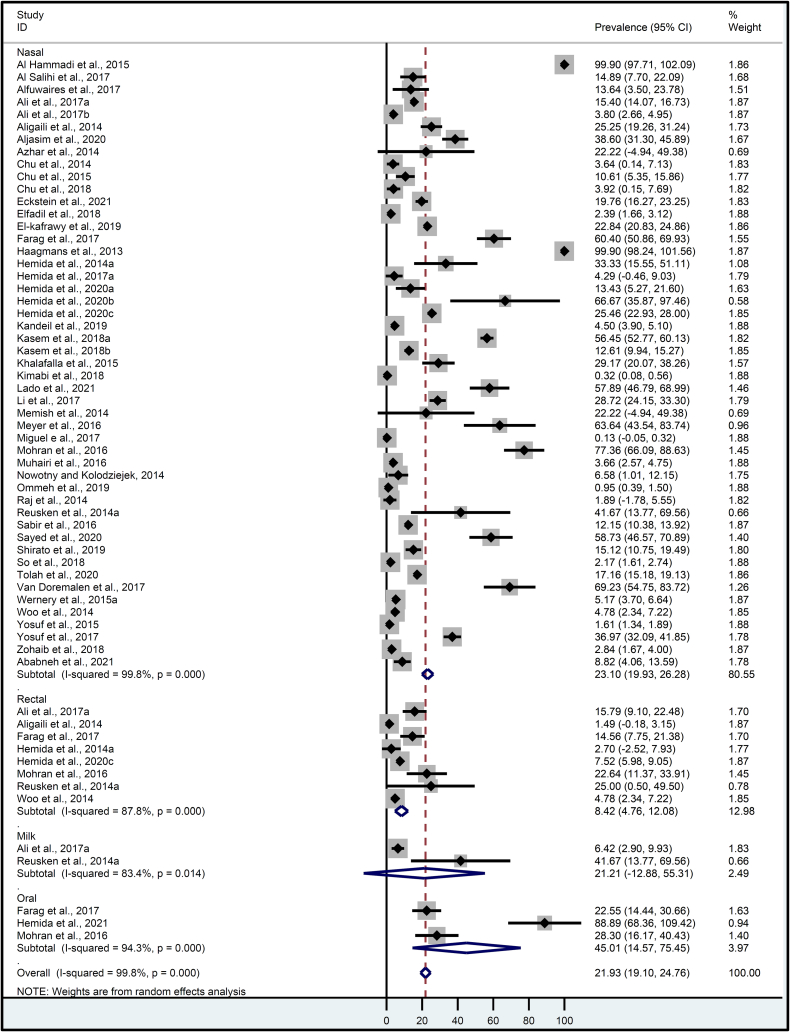
Forest plot showing the pooled MERS-CoV RNA prevalence in dromedary camels according to sample type. The central black square represents point estimates, whereas the square size represents the weight of each study in the meta-analysis.

**Fig. 8:**
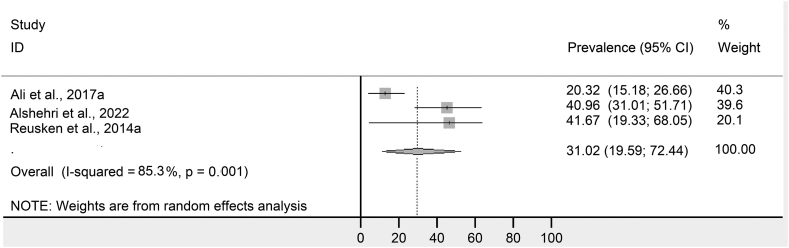
Forest plot showing the pooled prevalence of antibodies to MERS-CoV in milk of dromedary camels. The central black spot represents point estimates, whereas the square size represents the weight of each study in the meta-analysis.

Meta-analysis showed that the prevalence of MERS-CoV RNA varied significantly (*p* < 0.01) by sample type (Fig. 7). The pooled analysis detected that the highest viral RNA prevalence was in oral samples (45.1%, 95%CI: 14.57–75.45), followed by nasal (23.10%, 95%CI: 19.93–26.28), milk (21.21%, 95%CI: −12.88-55.31), and rectal (8.42%, 95%CI: 4.76–12.08) samples. Besides camel blood serum/plasma, camel milk was found to be positive for the antibodies to the virus (Fig. 8) with the estimated pooled prevalence of 31.02% (95%%CI: 19.59–72.44).

#### 3.4.2 Camel's age

**Fig. 9:**
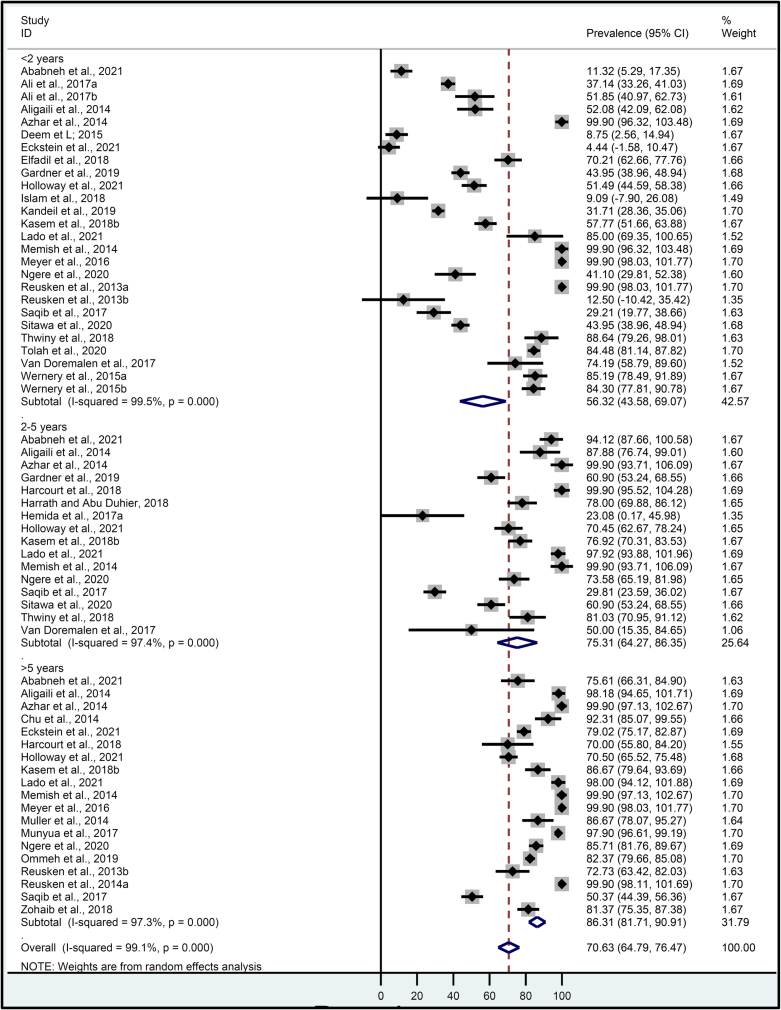
Forest plot showing the pooled seroprevalence of MERS-CoV in dromedary camels according to age. The central black square represents point estimates, whereas the square size represents the weight of each study in the meta-analysis.

**Fig. 10:**
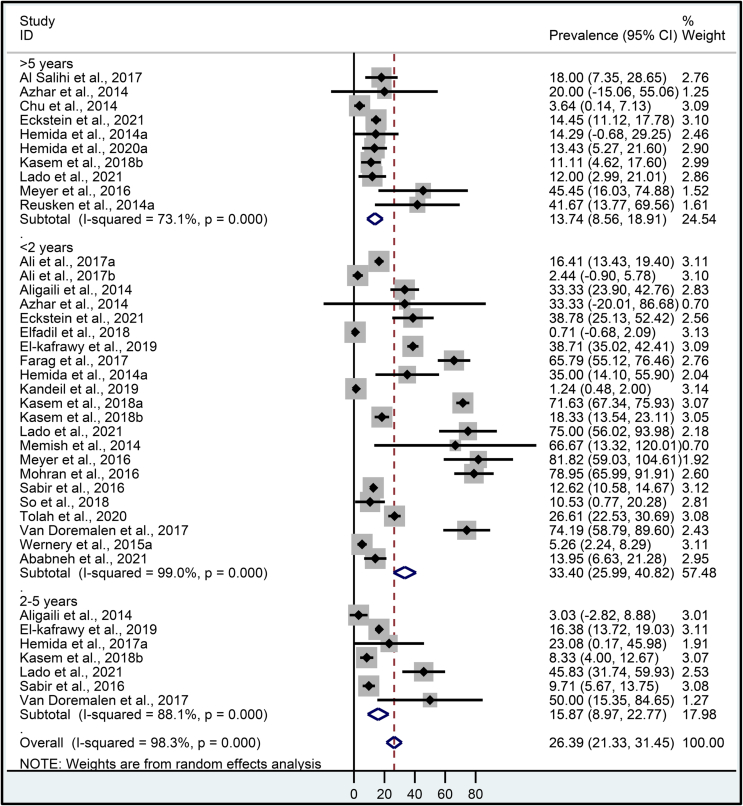
Forest plot showing the pooled MERS-CoV RNA prevalence in dromedary camels according to age. The central black square represents point estimates, whereas the square size represents the weight of each study in the meta-analysis.

While seroprevalence and prevalence of MERS-CoV RNA in DC varied significantly (*P* < 0.01) with age, the direction of age variation was inverse. For examples, seroprevalence was found to be lower in young camels and increased with age (Fig. 9). The seroprevalence in <2 years, 2–5 years, and > 5 years age camel groups were 56.32% (95%CI: 43.58–69.07), 75.31% (95%CI: 64.27–86.35), and 86.31% (95%CI: 81.71–90.91), respectively. On the other hand, the prevalence of viral RNA was higher in young camels and decreased with age (Fig. 10). The pooled analysis detected that the prevalence of viral RNA prevalence is 33.40% (95%CI: 25.99–40.82) in the <2 years old group, 15.87% (95%CI: 8.97–22.77) in the 2–5 years old group, and 13.74% (95%CI: 8.56–18.91) in the >5 years old group.

#### 3.4.3 Camel's sex

**Fig. 11:**
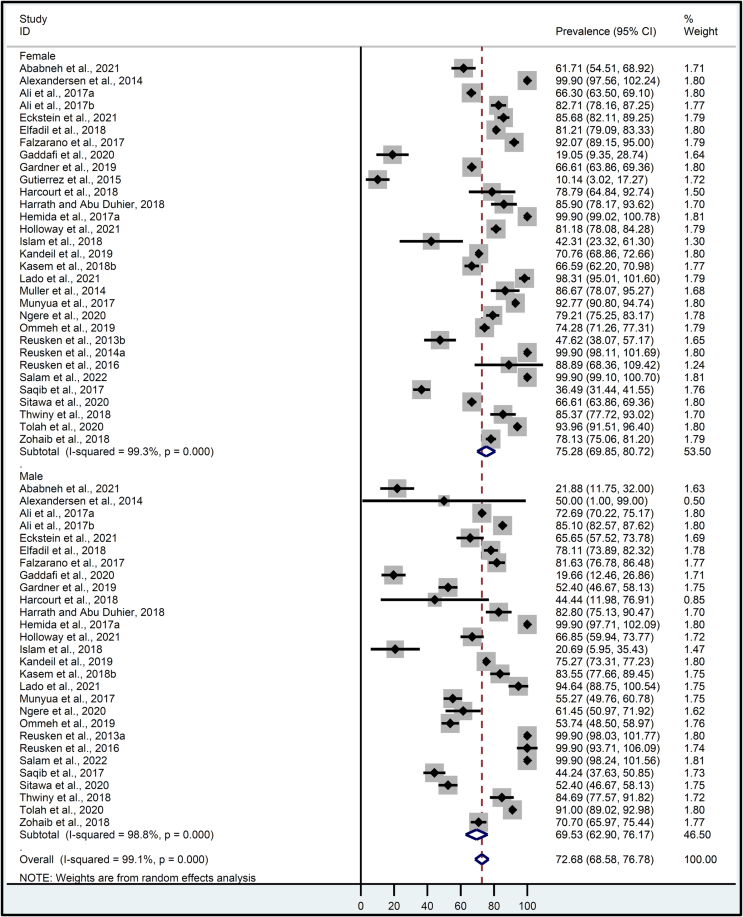
Forest plot showing the pooled seroprevalence of MERS-CoV in dromedary camels according to sex. The central black square represents point estimates, whereas the square size represents the weight of each study in the meta-analysis.

**Fig. 12:**
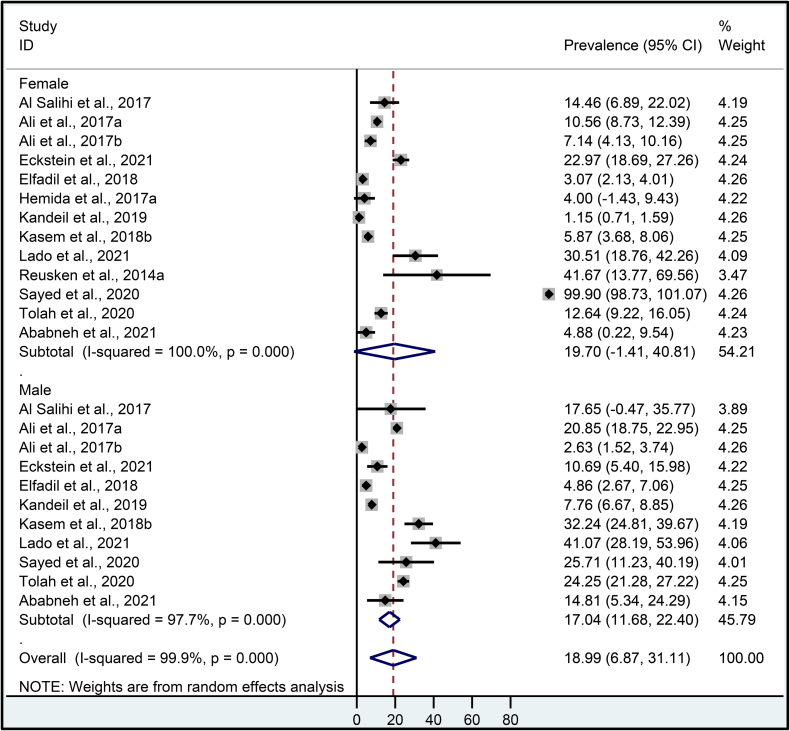
Forest plot showing the pooled MERS-CoV RNA prevalence in dromedary camels according to sex. The central black square represents point estimates, whereas the square size represents the weight of each study in the meta-analysis.

The estimated pooled prevalence of MERS-CoV varied significantly depending on the dromedary sex (*P* < 0.01) (Fig. 11 and Fig. 12). Seroprevalence was higher in females (75.28%, 95%CI: 69.85–80.72) than males (72.68%, 95%CI: 68.58–76.78). Similarly, viral RNA prevalence was higher in females (19.70%, 95%CI: −1.41-40.81) than males (17.04%, 95%CI: 11.68–22.40).

#### 3.4.4 Camel origin

**Fig. 13:**
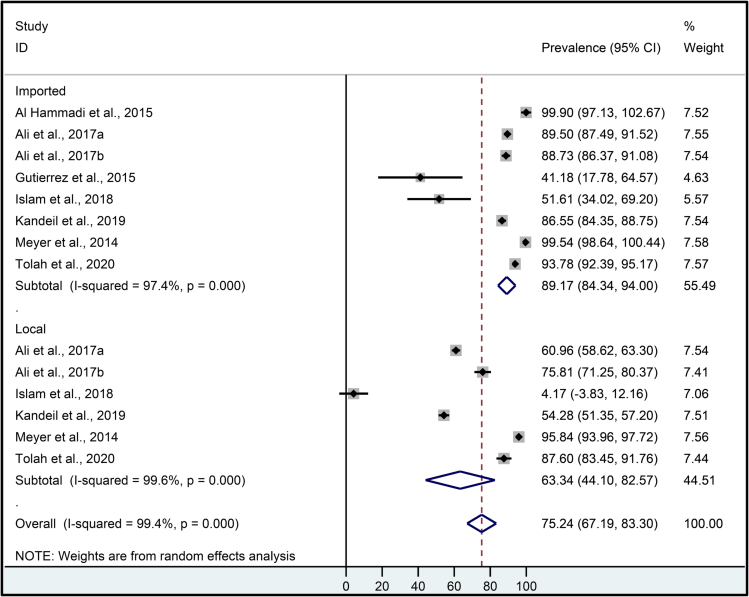
Forest plot showing the pooled seroprevalence of MERS-CoV in dromedary camels according to animal origin. The central black square represents point estimates, whereas the square size represents the weight of each study in the meta-analysis.

**Fig. 14:**
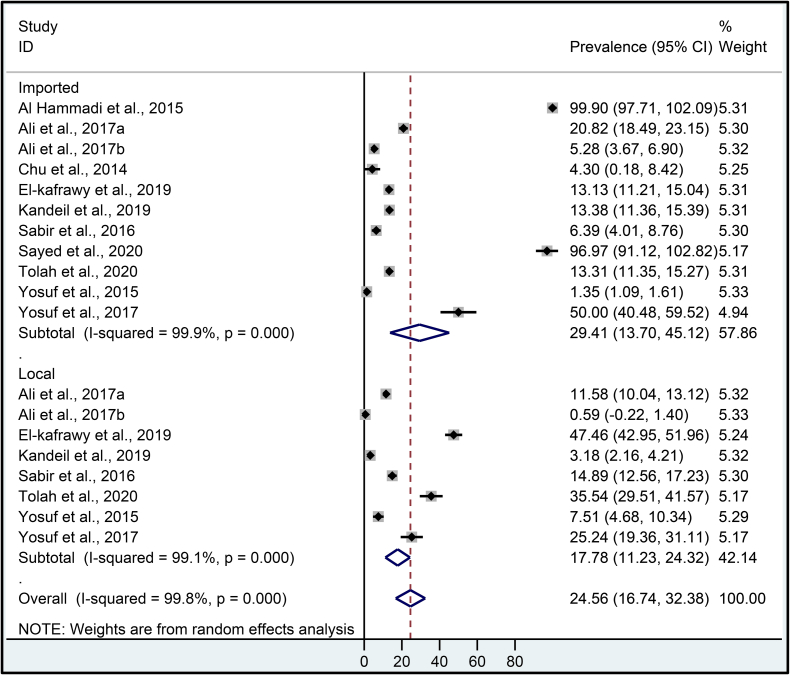
Forest plot showing the pooled MERS-CoV RNA prevalence in dromedary camels according to animal origin. The central black square represents point estimates, whereas the square size represents the weight of each study in the meta-analysis.

The origin of the camels was another factor that is associated with MERS-CoV prevalence (Fig. 13 and Fig. 14). Local camels had lower seroprevalence (63.34%, 95%CI: 44.10–82.57) and viral RNA prevalence (17.78%, 95%CI: 11.23–24.32) than those of imported camels (seroprevalence: 89.17%, 95%CI: 84.35–88.75 and viral RNA prevalence: 29.41%, 95%CI: 13.70–45.12).

#### 3.4.5 Camel management and sampling site

**Fig. 15:**
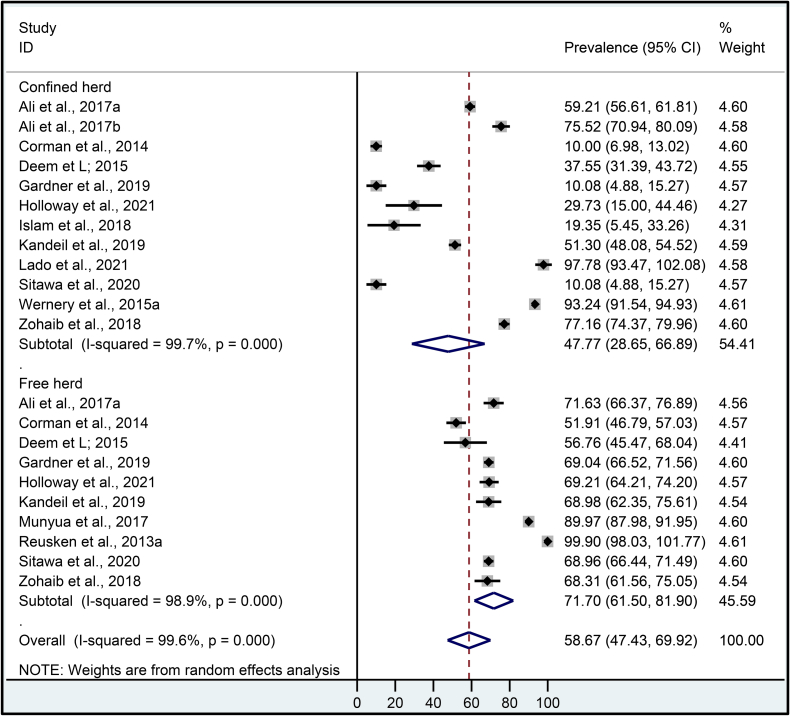
Forest plot showing the pooled seroprevalence of MERS-CoV in dromedary camels according to the camel management system. The central black square represents point estimates, whereas the square size represents the weight of each study in the meta-analysis.

Camel management was a significant factor in MERS-CoV prevalence (Fig. 15). Seroprevalence was higher among the camels of free-ranging herds (71.70%, 95%CI: 61.50–81.90) compared to those of confined herds (47.77%, 95%CI: 28.65–66.89).

**Fig. 16:**
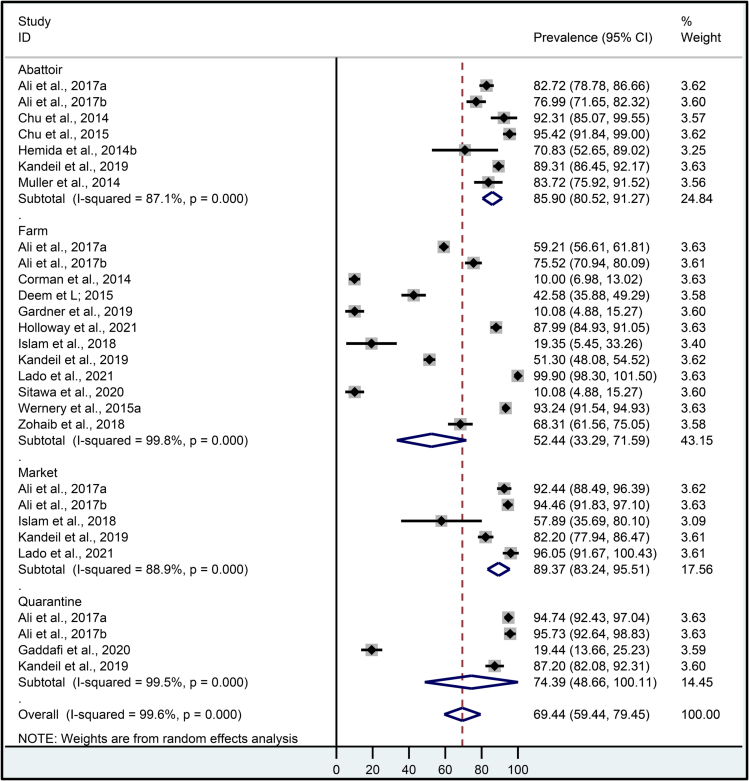
Forest plot showing the pooled seroprevalence of MERS-CoV in dromedary camels according to the sampling site. The central black square represents point estimates, whereas the square size represents the weight of each study in the meta-analysis.

**Fig. 17:**
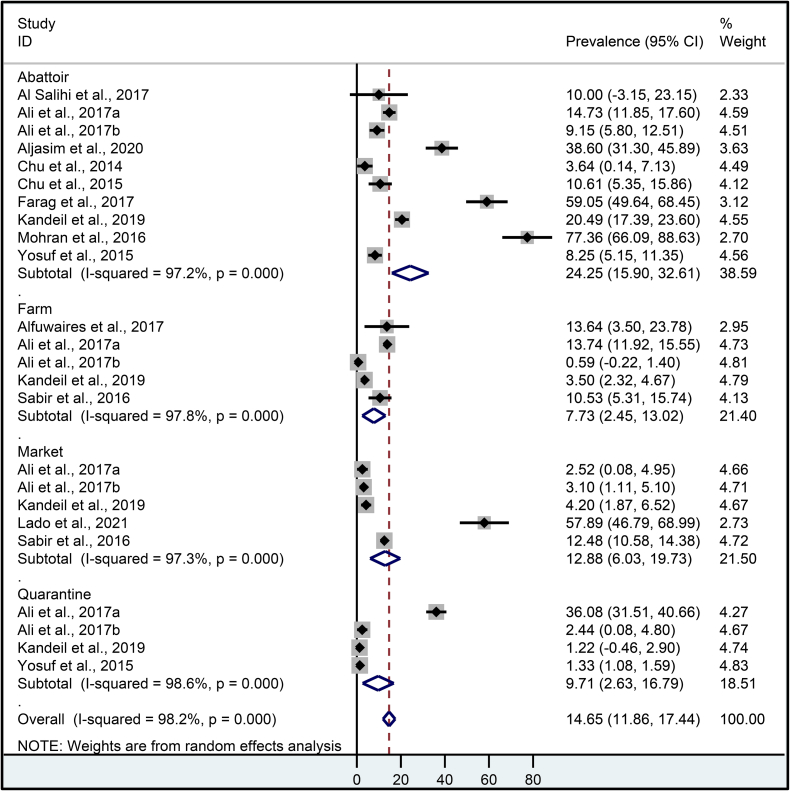
Forest plot showing the pooled MERS-CoV RNA prevalence in dromedary camels according to the sampling site. The central black square represents point estimates, whereas the square size represents the weight of each study in the meta-analysis.

The facility of sampling site was another factor that is associated with MERS-CoV prevalence and seroprevalence (Fig. 16 and Fig. 17). Seroprevalence was higher in samples that were collected from livestock markets, followed by abattoirs, quarantine, and farms with the rates of 89.37% (95%CI: 83.24–95.51), 85.90% (95%CI: 80.52–91.27), 69.44% (95%CI: 59.44–79.45), and 33.29% (95%CI: 33.29–71.59), respectively. The viral RNA prevalence was highest in samples collected from abattoirs (24.25%, 95%CI: 15.90–32.61), followed by livestock markets (12.88%, 95%CI: 6.03–19.73), quarantine (9.71%, 95%CI: 2.63–16.79), and farms (7.73%, 95%CI: 2.45–13.02).

#### 3.4.6 Other factors

We have reviewed articles in the current study and extracted data on modifiable risk factors that can influence MERS-CoV prevalence and seroprevalence in DC. However, the data for some of the extracted factors were insufficient to conduct a meta-analysis. Some articles showed that prevalence and seroprevalence could be changed due to differences in camel breed [24,25]. MERS-CoV seroprevalence was detected to be highest in the Majaheem breed (93.9%), followed by the Wadah (80.5%), Homor (80.3%), and Shaol (71.7%) breeds. The viral RNA prevalence was highest in Majaheem (2.6%) and Shaol (2.6%) breeds, followed by Homor (2.4%) and Wadah (2.2%%) breeds [25]. MERS-CoV prevalence was the highest during October (autumn) in Iraq [26], and during winter in Saudi Arabia [27]. Camel herd size, dung removal frequency, adding new camels to the herd, presence of nasal discharge, exposure to wild animals, and owners' ethnic background could affect MERS-CoV RNA and IgG prevalence [25,28,29]. One study reported viral RNA in semen [30], raising the possibility of sexual transmission of the virus.

## 4 Discussion

The analysis of archived serum of DC from Sudan and Somalia in 1983 was found to be seropositive to MERS-CoV [31], indicating that the virus was present in the dromedary population for at least 29 years before it caused clinical disease in humans in 2012 [4]. The virus was prevalent among DC since at least 1992 in Saudi Arabia [32]. Several studies were carried out to evaluate the presence of the virus in different animals, including DC, BC, NWC, sheep, goats, pigs, and horses [[32], [33], [34], [35]]. Only DC were identified with viral nucleic acid and antibodies to the virus. In Egypt and Jordan, sheep were found to be seropositive with low antibody titers without detecting the viral RNA [34,36], suggesting that these animals might had contracted the infection from camels, but the virus did not spread within the sheep host. Although BC and NWC are both vulnerable to the virus [[37], [38], [39]], and some of these animals were seropositive naturally [[40], [41], [42], [43]], no viral RNA was found in BC and NWC through natural infection, suggesting that only DC may be involved in viral transmission in naturally.

There are 46 countries where camels can be found in their natural habitat. DC are predominant in North Africa, East Africa, West Africa, West Asia, and South Asia [44]. The virus may be prevalent only in these regions, that is why the viral nucleic acid was detected in DC of these regions. No nucleic acid analysis for MERS-CoV was positive in the camels from rest of the world. Seropositive camels found in the Canary Islands were imported from Africa long years back [45]. Similarly, the seroprevalence among DC and BC in Israel, Mongolia and Kazakhstan [40,41,46] may be due to any link and introduction of the virus from the prevalent regions, which needs more investigation.

According to the meta-analysis, the global seroprevalence of MERS-CoV in DC is 77.4%, consistent with earlier research [19], which reported pooled seroprevalence of 73% and 83.9% by ELISA and Immunofluorescent antibody tests, respectively. The current study found that MERS-CoV prevalence and seroprevalence among the dromedaries of the Western Asia are higher than those of the rest of the world, which is supported by previous studies [14]. Variation in prevalence and seroprevalence among camels of different regions of the world may be due geo-climatic variability, breed variation, camel density, husbandry, management and movement pattern [14,[47], [48], [49]].

MERS-CoV is considered to be endemic in camels in the Arabian Peninsula [13], where DC, beside livestock, are also used for racing and beauty contest. Camels for racing and beauty contest have an incredibly high value. Consequently, the management system of such camels in this region differs from rest of the world [49,50]. The camel density and movement way in this region are also different [14,48,49,51]. UAE, Qatar, and Kuwait are the countries with the highest camel population density globally [14]. While animal grazing in the deserts of Qatar is forbidden [52,53], camels from Qatar travel to Saudi Arabia during the season of grazing and breeding. In addition, racing camels travel between the Arabian Gulf countries during camel racing and beauty contest without restricting cross border movement. Often Qatari livestock farms house camels in a small area with several other animals and birds, where the animal attendants also share the same premises. Animal owners have resting place (majlis) inside the farm premise, where they spend their leisure time. There is not human or animal movement control inside these farms. In the racing camel farms in Qatar, although no additional livestock are present, camels are kept in high density without biosecurity practice. During racing season, these farms host camels from other Gulf countries without any quarantine practice. A similar picture of camel movement and management is seen in the other countries of the Arabian Peninsula. Knowledge, attitude, and practices by the native camel owners can be another issue of high prevalence of MERS-CoV in camels of the Western Asia. The local people are usually reluctant to follow the MERS-CoV control related recommendations and regulations due to the perceived low risk associated with MERS-CoV [54].

Several studies were conducted for MERS-CoV nucleotide confirmation using nasal, oral, milk, rectal, urine, and semen samples, with urine samples consistently negative for viral RNA detection [55]. Most evaluated studies used nasal samples as the recommended sample of choice [56] for MERS-CoV confirmation. In this investigation, we found that the prevalence of MERS-CoV RNA in oral samples was higher than in nasal samples, which could be owing to the smaller sample size of oral samples in the current study's pooled prevalence investigation. We propose, however, that oro-nasal samples be used to confirm MERS-CoV in camels.

Similar to our study, a previous systematic review [14] supports the finding that seroprevalence and viral prevalence increase and decrease with DC age, respectively. Our study proves the findings by meta-analysis with a weight of 100%. Age-dependent seroprevalence can be due to the higher prevalence of viral shedding in juveniles than in adults and is likely due to immunological naivety [57,58]. Our study's estimated pooled seroprevalence and viral RNA prevalence showed that MERS-CoV virus is more prevalent in female camels. The extracted data were insufficient to conduct analysis combining age and sex. Therefore, it was not possible to assess whether sex is a factor in camels of all age groups. In general, camel gender may not be a factor in seroprevalence or viral RNA prevalence in young camels. In adult camels, however, the seroprevalence and viral RNA prevalence of female camels might be higher than those of male camels. It is evident that mother camels are more in contact with their young, which have higher viral load and shedding, and hence more likely to get infections from their offspring.

Travel is a dynamic process of infectious disease transmission globally [59,60]. In addition, camel trade is an important route for introducing a virus into importing farms/countries [35]. An animal can be transported from one country to another during the incubation period or get a new infection from other infected animals during transportation. Transportation generates stress, thus lowering infection resistance. This may be why imported camels have always had a greater prevalence and seroprevalence of MERS-CoV than native camels. Camels at abattoir, market, quarantine, and herd type follow the same principles. When camels are in the market or waiting to be slaughtered in the abattoir, they are likely to contact camels from different areas, making them prone to illness due to transportation stress. This may be the cause that camels of abattoir, market, or quarantine camels are more prevalent for MERS-CoV than farm camels. Free herd camels, such as pastoralist or nomadic camels, are more exposed to outside camels than those of confined herds. As a result, free herd camels have a higher prevalence than confined herd camels.

Prevention and control of the virus in DC can be the best way to reduce the risk of infection at the human-animal interface. As the virus is more likely to infect the young camels, and these young camels act as a source of viral shedding [58], the viral spread should be halted while the camel is still young. Vaccination could help MERS-CoV infection control, and the young camels should be given higher consideration for vaccination. However, no licensed MERS-CoV vaccine is currently available for camels (or humans) [61].

## 5 Conclusions

The findings of our study show that MERS-CoV is mainly distributed among dromedary camels in the Asian and African regions. Highest estimated pooled prevalence and seroprevalence were detected in West Asia. Risk factors such as sample type, young age, female sex, imported camels, and camel management must be considered to control and prevent MERS-CoV. Vaccination of camels, public awareness, camel farm management and biosecurity, and camel movement control are necessary to reduce MERS-CoV spreading.

## Funding

No funding was received for this study.

## Declaration of Competing Interest

No conflict of interest to declare.

## Data Availability

The work was done with publicly available data. There is no additional data to make open for the readers.

## Appendix A Supplementary data

Supplementary material 1 Image 1

Supplementary material 2 Image 2

Supplementary material 3 Image 3

Supplementary material 4 Image 4
