# Supplementary material for: Global patterns of Middle East respiratory syndrome coronavirus (MERS-CoV) prevalence and seroprevalence in camels: A systematic review and meta-analysis
Source: One Health. 2023 May 8;16:100561. doi: 10.1016/j.onehlt.2023.100561 (PMC10166617; doi:10.1016/j.onehlt.2023.100561)
Supplement: Supplementary file 3 — Supplementary material 3 [file mmc3.pdf]

Supplementary file 3: Extracted data from the selected articles

Ref	Author	Sampling country, time and animal	Molecular study results	Immunologic study results	Additional information	Remark
1	Ababneh et al., 2021[1]	Jordan; 2018-2020; DC	Nasal: 136/16<br>Male: 54/9<br>Female: 82/7<br><2 years: 106/16<br>> 5 years: 30/0<br>Winter: 46/16	Serum: 239/130<br>Male: 64/16<br>Female: 175/114<br><2 years: 106/20<br>2-5 years: 51/45<br>>5 years: 85/65		All samples were included in meta-analysis.<br>Quality: Intermediate
2	Al Hammadi et al., 2015[2]	UAE; 2015; DC	Nasal/Spring/Imported: 21/13<br><2 years: 12/8<br>>5 years: 3/2<br>Female: 18/8	Serum/Spring/Imported: 10/8<br><2 years/female: 10/7<br>>5 years: 2/1	• The positive animals had mild to mucopurulent nasal discharge<br>• After seven days of initial positive, 5 animals were positive<br>• Two young dromedaries had high viral load and lowest neutralizing antibodies	All samples were included in meta-analysis.<br>Quality: High
3	Al Salihi et al., 2017[3]	Iraq; 2015-2016; DC	Nasal: 94/14<br>Oropharyngeal: 6/1<br><2 years: 9/0<br>>5 years: 50/9<br>Female: 83/12<br>Male: 17/3<br>Autumn: 56/4<br>Winter: 80/7<br>Abattoir: 20/2		• There was no significant difference in prevalence between sex and sample type,<br>• Aged animals had high prevalence than young animals.<br>• Study location and season have significance on prevalence of the virus. October was the highest prevalent season.	PCR method was considered for analysis in the current study<br>Quality: High
4	Alagaili et al., 2014[4,5]	Saudi Arabia; 1992-2013; DC	Nasal: 202/51<br>Rectal: 202/3<br><2 years: 96/32<br>2-5 years: 33/1<br>>5 years: 55/0	Serum: 467/380<br><2 years: 96/50<br>2-5 years: 33/29<br>>5 years: 55/54	• The seroprevalence was higher in the adults than in juveniles, where the viral RNA prevalence was higher in juveniles than adults	Quality: High
5	Alexandersen et al., 2014[6]	UAE; 2005<br>USA and Canada; 2000-2001; DC		Serum: 17/9<br>UAE: 11/9<br>North America: 6/0<br>Female: 7/7<br>Male: 4/2	• MERS-CoV or its closely related virus was not new in camels in the Middle East during 2005.	Quality: Intermediate
6	Alfuwaires et al., 2017[7]	Saudi Arabia; 2015; DC	Nasal/farm: 44/6	Serum: 124/0<br>Abattoir: 78/0<br>Farm: 44/0	• The role of camels as hosts of MERS-CoV is not well understood in this research<br>• Sample source: dystocia, respiratory distress, mastitis, fracture, fever, general health check.	Quality: High
7	Ali et al., 2017a[8]	Egypt; 2014-2016; DC	Nasal: 2825/435<br>Rectal: 114/18<br>Milk: 187/12<br><2 years: 591/97<br>Female: 1089/115<br>Male: 1439/300<br>Abattoir: 584/86<br>Farm: 1376/189<br>Market: 159/4<br>Quarantine: 424/153<br>Imported: 1167/243<br>Local: 1658/192	Milk: 187/38<br>Serum: 2541/1808<br>Market: 172/159<br>Free herd: 282/202<br>Farm: 1373/813<br>Quarantine: 361/342<br>Abattoir: 353/292<br><2 years: 595/221<br>Male: 1245/905<br>Female: 1092/724<br>Imported: 886/793<br>Local: 1665/1015	• Seroprevalence and viral nucleic acid prevalence can differ due to sampling location, age, sex, and camel origin.<br>• Antibodies rapidly go down, and camels get reinfected, and the outbreak in a herd is sustained for an extended time<br>• Maternal antibody titer level is very low in calves regardless of the mother's antibody titer.<br>• Camel trade is an important route of introducing the virus into importing countries	Quality: High
8	Ali et al., 2017b[9]	Egypt; 2015-2016; DC	Nasal swab: 1078/41<br>Milk: 38/0<br>Rectal: 13/0<br><2 years: 82/2<br>Male: 798/21<br>Female: 280/20<br>Abattoir: 284/26<br>Farm: 340/2<br>Market: 290/9<br>Quarantine: 164/4<br>Local: 340/2<br>Imported: 738/39	Serum: 1031/871<br>Local: 339/257<br>Imported: 692/614<br>Market: 289/273<br>Farm: 339/256<br>Quarantine: 164/157<br>Abattoir: 239/184<br><2 years: 81/42<br>Male: 765/651<br>Female: 266/220	• Sample origin and age of the animals had significant associations with seroprevalence of MERS-CoV in camels;<br>• Age and sample origin are also significantly associated with viral RNA	Quality: High
9	Aljasim et al., 2020[10]	Saudi Arabia; 2019; DC	Nasal/abattoir: 171/66	Serum/abattoir: 161/114	• The study showed high prevalence of the virus and seroprevalence among camels in slaughterhouse during the study	Quality: High
10	Alshehri et al., 2022[11]	Saudi Arabia; 2020; DC	Milk: 83/7	Milk: 83/34	• Antibody prevalence in milk was influenced by region, camel breed, and age	Quality: High

Ref	Author	Sampling country, time and animal	Molecular study results	Immunologic study results	Additional information	Remark
					• Viral RNA was influenced by region, and camel breed.	
11	Azhar et al., 2014[12]	Saudi Arabia; 2013; DC	Nasal: 9/2<br><2 years: 3/1<br>2-5 years: 1/0<br>>5 years: 5/1<br>Autumn: 9/2	Serum: 9/9<br><2 years: 3/3<br>2-5 years: 1/1<br>>5 years: 5/5<br>Autumn: 9/9	• The study showed evidence of camel-to-human transmission of MERS-CoV	Quality: Intermediate
12	Bold et al., 2021[13]	Mongolia; 2016- 2017; BC		Serum: 180/17		Quality: Intermediate
13	Chan et al., 2015[14]	Mongolia; 2014; BC	Nasal: 200/0	Serum: 200/0		Quality: High
14	Chu et al., 2014[15]	Egypt; 2013; DC	Nasal/>5 years: 110/4<br>Local/Farm: 17/0<br>Imported/Abattoir: 93/4	Serum/abattoir/>5 years: 52/48		Quality: Intermediate
15	Chu et al., 2015[16]	Nigeria, 2015; DC	Nasal/Winter/Abattoir: 132/14	Serum/Winter/Abattoir: 131/125		Quality: Intermediate
16	Chu et al., 2018[17]	Ethiopia; 2017; DC	Nasal: 102/4			Quality: High
17	Corman et al., 2014[18]	Kenya; 1992-2013; DC		Serum: 774/228<br>Free herd: 366/190<br>Farm: 408/38	• Nomadic camels were more prevalent than the farmed camels<br>• Adults had higher seroprevalence than Youngs<br>• The virus might be correlated with camel population density.	Nomadic was considered as free herd camels.<br>Quality: Intermediate
18	Crameri et al., 2015[19]	Australia; 2013-2014; DC		Serum: 307/0<br>Abattoir:231/0<br>Free herd: 76/0<br>Summer: 131/0<br>Autumn: 100/0<br>Winter: 76/0		Feral camels were considered free herds.<br>Quality: High
19	David et al., 2018[20]	Israel; 2012-2017;<br>Llama, Alpaca, DC	DC/Nasal: 540/0<br>Alpaca/Nasal: 102/0<br>Llama/Nasal: 19/0	DC/serum: 411/254<br>Alpaca/serum: 102/35<br>Llama/serum: 19/7	• Although the viral antibody is available in this country, there is no active circulation of the virus in Israel.	ELISA was used in meta-analysis.<br>Quality: Intermediate
20	Deem et al., 2015[21,22]	Kenya; 2013; DC		Serum: 335/166<br><2 years: 80/17<br>Farm: 209/89<br>Free herd: 126/69<br>Confined herd: 237/89	• Adult camels were more seroprevalent than youngs	Nomadic and pastoralist camels were considered free herds, and others were considered farm camels.<br>Quality: High
21	Eckstein et al., 2021[23]	Tunisia; 2020; DC	Nasal/Winter: 501/99<br>Male: 131/14<br>Female: 370/85<br><2 years: 49/19<br>>5 years: 429/62	Serum/Winter: 501/403<br>Male: 131/86<br>Female: 370/317<br><2 years: 45/2<br>>5 years: 429/339		Quality: High
22	Elfadil et al., 2018[24]	Saudi Arabia; 2015; DC	Nasal/Spring: 1673/40<br><2 years: 141/1<br>Male: 370/18<br>Female: 1303/40	Serum: 1674/1348<br><2 years: 141/99<br>Male: 370/289<br>Female: 1304/1059	• Sampling location, camel breed and age, grazing system, herd size, dung removal frequency, adding new animals, presence of nasal discharge, and exposure to wild animals are significantly involved in MERS-CoV seroprevalence in camels.<br>• Sampling location, grazing system, camel sex, herd size, dung removal frequency, and nasal discharge are significant factors of MERS-CoV RNA prevalence in camels	Quality: High
23	El-Kafrawy et el., 2019[25]	Saudi Arabia 2016-2018; DC	Nasal: 1668/381<br>Local: 472/224<br>Imported: 1196/157<br><2 years: 669/259<br>2-5 years: 745/122		• Viral RNA prevalence varied among imported and local animals and different aged animals.	Quality: High
24	Falzarano et al., 2017[26]	Mali, 2009 to 2010; DC		Serum/local: 570/502<br>Male: 245/200<br>Female: 328/302		Quality: High
25	Farag et al., 2019[27]	Qatar, 2015; DC	Nasal: 101/61<br>Oral: 102/23<br>Rectal: 103/15<br>Bronchial: 101/7	Serum: 103/100		Quality: High

Ref	Author	Sampling country, time and animal	Molecular study results	Immunologic study results	Additional information	Remark
			Lymph node: 53/5<br><2 years: 76/50<br>Abattoir: 105/62			
26	Gaddafi et al., 2020[28]	Nigeria; 2016; DC		Serum: 180/35<br>Female: 63/12<br>Male: 117/23<br>Quarantine: 180/35		Quality: High
27	Gardner et al., 2019[29]	Kenya; 2017; DC		Serum: 1421/905<br><2 years: 380/167<br>2-5 years: 156/95<br>Female: 1129/752<br>Male: 292/153<br>Free herd: 1292/892<br>Farm: 129/13		Quality: High
28	Gutierrez et al., 2015[30]	Canary Island; 2015; DC		Serum: 170/7<br>Local: 153/0<br>Imported: 17/7<br>Male: 101/0<br>Female: 69/7	• All the seropositive camels were imported from Africa 20 or more years ago	Quality: High
29	Haagmans et al., 2014[31]	Qatar; 2013; DC	Nasal/Autumn: 14/14			Quality: High
30	Harcourt et al., 2018[32]	Israel; 2013; DC		Serum: 71/31<br>2-5 years: 2/2<br>>5 years: 40/28<br>Male: 9/4<br>Female: 33/26	• There is no association of age or sex with seroprevalence	Quality: Intermediate
31	Harrath and Abu Duhier, 2018[33]	Saudi Arabia; 2016; DC		Serum: 171/144<br>Male: 93/77<br>Female: 78/67<br>2-5 years: 100/78	• Seroprevalence is significantly associated with age but not related to sex or origin of animals	Quality: High
32	Hasan et al., 2013 [34]	Iraq; 2017-18; DC		Serum: 171/31<br>2-5 years: 2/2<br>>5 years: 72/65<br>Female: 40/36<br>Male: 50/45	•	Quality: Intermediate
33	Hemida et al., 2013[35]	Saudi Arabia; 2012-2013; DC		Serum: 310/280<br><2 years: 65/47<br>2-5 years: 76/74<br>>5 years: 63/58		Quality: Intermediate
34	Hemida et al., 2014a[36]	Saudi Arabia; 1993; DC<br>Egypt; 2014; DC<br>Australia; 2014; DC		Serum:163/125<br>Saudi Arabia: 131/118<br>Egypt: 7/7<br>Australia: 25/0<br>Free herd: 17/0<br>Abattoir: 24/17<br>Farm: 8/0		Feral camels were considered free herds. Quality: Intermediate
35	Hemida et al., 2014b[37]	Saudi Arabia; 2013 to 2014; DC	Nasal: 27/9<br>Oral: 14/0<br>Rectal: 37/1<br><2 years: 20/7<br>>5 years: 21/3			Quality: Intermediate
36	Hemida et al., 2017[38]	Saudi Arabia; 2014- 2015; DC	Nasal: 221/3<br>Rectal: 222/0<br>Female: 193/2<br>Male: 19/0<br><2 years: 8/0<br>2-5 years: 55/3<br>>5 years: 131/0<br>Autumn: 78/3<br>Spring: 94/0<br>Winter: 51/0	Serum: 465/465<br>Female: 347/347<br>Male: 46/46<br><2 years: 55/55<br>2-5 years: 91/91<br>>5 years: 229/229<br>Autumn: 84/84<br>Winter: 94/94		Every sample was included in meta-analysis. Quality: High
37	Hemida et al., 2020a[39]	Saudi Arabia; 2015-2017; DC	Adult male semen: 67/9			Quality: High
38	Hemida et al., 2020b[40]	Saudi Arabia; 2019; DC	Nasal/Spring: 27/10<br>Breath: 27/6			Every sample was included meta-analysis. Quality: High
39	Hemida et al., 2020c[41]	Saudi Arabia; 2016- 2018; DC	Nasal/abattoir: 1131/288<br>Local: 764/203<br>Imported: 367/85<br>Rectal: 1131/85			Quality: High
40	Hemida et al., 2021[42]	Saudi Arabia; 2019; DC	Oral: 9/8<br>Conjunctiva: 9/3			Quality: High

Ref	Author	Sampling country, time and animal	Molecular study results	Immunologic study results	Additional information	Remark
41	Holloway et al., 2021[43]	Jordan; 2014-2018; DC	Nasal/rectal: 837/0	Serum/farm: 837/745<br><2 years: 202/104<br>2-5 years: 132/93<br>>5 years: 322/290<br>Male: 178/119<br>Female: 611/496<br>Free herd: 328/227<br>Close herd: 37/11<br>Winter: 247/178<br>Spring: 127/95<br>Summer: 185/168<br>Autumn: 230/174	• Seroprevalence among females was more than males, open herd than close herd	Quality: High
42	Islam et al., 2018[44]	Bangladesh; 2015; DC	Nasal/Rectal: 55/0	Serum/Autumn: 55/17<br>Farm: 31/6<br>Market: 19/11<br>Imported: 31/16<br>Local: 24/1<br><2 years: 11/1<br>Male: 29/6<br>Female: 26/11	• Adults and imported camels were more prevalent than juvenile and local camels, respectively.	Quality: High
43	Kandeil et al., 2019[45]	Egypt; 2016-2018<br>Senegal; 2017; DC<br>Tunisia; 2015 to 2018; DC<br>Uganda; 2017<br>Iraq; 2017; DC<br>Saudi Arabia; 2017; DC<br>Jordan; 2015 to 2016; DC	Nasal: 4581/206<br>Egypt: 2230/183<br>Senegal: 127/7<br>Uganda: 500/0<br>Tunisia: 1170/9<br>Saudi Arabia: 222/0<br>Jordan: 304/0<br>Iraq: 26/0<br>Male: 2319/180<br>Female: 2262/26<br><2 years: 808/10<br>Market: 286/12<br>Farm: 944/33<br>Quarantine: 164/2<br>Abattoir: 649/133<br>Local: 1131/36<br>Imported: 1099/147<br>Winter: 248/0	Serum: 4071/2962<br>Egypt: 2033/1401<br>Senegal: 198/129<br>Uganda: 500/308<br>Tunisia: 782/683<br>Saudi Arabia: 222/205<br>Jordan: 304/246<br>Iraq: 32/14<br>Male: 1860/1400<br>Female: 2206/1561<br><2 years: 741/235<br>Market: 309/254<br>Free herd: 187/129<br>Farm: 924/474<br>Quarantine: 164/143<br>Abattoir: 449/401<br>Local: 1111/603<br>Imported: 922/798<br>Winter: 254/219	• Seroprevalence differed significantly by sample collection site, animal age, sex, and origin	Quality: High
44	Karamendin et al., 2022[46]	Kazakhastan; 2020-21, DC and BC		Serum DC: 120/0<br>Serum BC: 129/0		Quality: Intermediate
45	Kasem et al., 2018a[47]	Saudi Arabia; 2015-2017; DC	Nasal: 698/394<br>Winter: 537/384<br>Summer: 161/10<br><2 years: 423/303		• The viral RNA prevalence was significantly influenced by sampling location, season of sampling and age of the animals.	Rapid test was not considered for analysis. Quality: High
46	Kasem et al., 2018b[48]	Saudi Arabia; 2014-2016; DC	Nasal: 595/75<br>Male: 152/49<br>Female: 443/26<br><2 years: 251/46<br>2-5 years: 156/13<br>>5 years: 90/10	Serum: 595/422<br>Male: 152/127<br>Female: 443/295<br><2 years: 251/145<br>2-5 years: 156/120<br>>5 years: 90/78	• Viral RNA prevalence was significantly influenced by sampling region, sex, and age of the camels.<br>• Seroprevalence was influenced by sex and age of camels.	Quality: High
47	Khalfalla et al., 2015[49]	Saudi Arabia; 2013 to 2014; DC	Nasal/Spring: 96/28			Quality: High
48	Kiambi et al., 2018[50]	Kenya; 2016 to 2017; DC	Nasal: 2175/7			Quality: High
49	Kim et al., 2016[51]	Korea; June 2015; DC, BC	DC and BC: 46/0			Quality: High
50	Lado et al., 2021[52]	UAE; 2019; DC	Nasal: 76/44<br>Farm: 15/0<br>Market: 76/44<br>Confined herd: 45/0<br>Female: 59/18<br>Male: 56/23<br><2 years: 20/15<br>2-5 years: 48/22<br>>5 years: 50/6	Serum: 121/107<br>Farm: 15/15<br>Market: 76/73<br>Confined herd: 45/44<br>Female: 59/58<br>Male: 56/53<br><2 years: 20/17<br>2-5 years: 48/47<br>>5 years: 50/49		ELISA was considered for analysis. Quality: High
51	Lau et al., 2020[53]	UAE; 2019; BC, HC<br>China; 2012; BC		UAE/BC/Serum/Spring: 29/14<br>UAE/HC/Serum: 11/6<br>China/BC/Serum: 92/0		ELISA was considered for analysis. Quality: High
52	Li et al., 2017[54]	UAE; DC; 2015	Nasal: 376/108			Quality: High

Ref	Author	Sampling country, time and animal	Molecular study results	Immunologic study results	Additional information	Remark
53	Liu et al., 2015[55]	Mongolia; 2015; BC	Nasal: 190/0	Serum: 190/0		Quality: High
54	Memish et al., 2014[56]	Saudi Arabia; 2013; DC	Nasal/Autumn: 9/2<br><2 years: 3/2<br>2-5 years: 1/0<br>>5 years: 5/0	Serum/Autumn: 9/9<br><2 years: 3/3<br>2-5 years: 1/1<br>>5 years: 5/5	• Evidence that camel is a source of human MERS-CoV infection	Quality: High
55	Meyer et al., 2014[57]	UAE; 2003-2013; DC, BC<br>Germany; 2003-2013; BC		DC/Serum: 651/632<br>DC/Imported: 218/217<br>DC/Local: 433/415<br>BC/Germany/Serum: 16/0		Quality: High
56	Meyer et al., 2016[58]	UAE; 2014-2015; DC	Nasal: 22/14<br><2 years: 11/9<br>>5 years/female: 11/5	Serum: 22/22<br><2 years: 11/11<br>>5 years/female: 11/11		Adults (mothers) and youngs were considered as >5 years and <2 years group respectively. Quality: Intermediate
57	Miguel et al., 2016[59]	Kazakhstan; 2015; DC, BC		DC/Female/Serum: 550/0<br>BC/Female/Serum: 95/0		Quality: High
58	Miguel et al., 2017[60]	Burkina faso, Ethiopia, Morocco; 2015; DC	Nasal: 1500/2<br>Burkina Faso: 525/1<br>Ethiopia: 632/1<br>Morocco: 343/0	Serum: 1500/1251<br>Burkina Faso: 525/414<br>Ethiopia: 632/583<br>Morocco: 343/254		Calculated from % values. Quality: High
59	Moazemi-Goudarzi et al., 2022[61]	Iran, 2014; DC	Nasal/Rectal: 18/3		These animals were illegally transported from Pakistan to Iran.	Quality: High
60	Mohran et al., 2016[62]	Qatar; 2014; DC	Nasal/Winter/Abattoir: 53/41<br>Oral: 53/15<br>Rectal: 53/12<br><2 years: 38/30			Quality: High
61	Muhairi et al., 2016[63]	UAE; 2014; DC	Nasal: 1147/42			Quality: High
62	Müller et al., 2014[64]	Egypt: 1997; DC<br>Somalia: 1983-1984; DC<br>Sudan: 1983; DC		Serum/Summer: 189/159<br>Egypt: 43/35<br>Somalia/Abattoir:86/72<br>Sudan/Female/>5 years: 60/52		ELISA was used for analysis. Quality: High
63	Munyua et al., 2017[65]	Kenya; 2013; DC		Serum/Free herd: 877/789<br>Female: 664/616<br>Male: 213/173<br>>5 years: 476/466	• Female camels are more prevalent than male camels<br>• Aged camels are more prevalent than young camels	Quality: High
64	Ngere et al., 2020a[66]	Kenya; 2018; DC		Serum: 498/376<br><2 years: 73/30<br>2-5 years: 106/78<br>>5 years: 301/258<br>Female: 404/320<br>Male: 83/51	• Seroprevalence differs due to camel sex, owners' ethnic background, and camel herd size.	Quality: High
65	Ngere et al., 2020b[67]	Kenya; 2018; DC	Nasal: 4692/124			Quality: High
66	Nowotny and Kolodziejek, 2014[68]	Oman; 2013; DC	Nasal and Conjunctival/Winter: 76/5			Quality: Intermediate
67	Ommeh et al., 2019[69,70]	Kenya; 2016 to 2018; DC	Nasal: 1163/11	Serum: 1163/801<br>Male: 348/187<br>Female: 801/595<br>>5 years: 760/626	• Age and sex of the camels and location of sample collection are significantly associated with MERS-CoV seroprevalence.<br>• Adults are more serorevalent than youngs	Quality: High
68	Orynbayev et al., 2022[71]	Kazakhastan; 2017-2018; BC and DC	DC Nasal: 1534/0<br>BC Nasal: 963/0	DC Serum: 5083/17<br>BC Serum: 3123/12	• Seroprevalence was higher among adults than young and females than males	Quality: High
69	Perera et al., 2013[72]	Egypt; 2013; DC		Serum/Summer: 110/103		Quality: High

Ref	Author	Sampling country, time and animal	Molecular study results	Immunologic study results	Additional information	Remark
70	Raj et al., 2014[73]	Qatar; 2014; DC	Nasal/Winter: 53/1			Quality: High
71	Reusken et al., 2013a[74]	Jordan; 2013; DC		Serum/ Male/<2 years: 11/11		Outdoor management has been considered free herd.<br>Quality: High
72	Reusken et al., 2013b[75]	Canary Island: April 2012 to May 2013; DC<br>Oman; March 2013; DC<br>Chile; 2011; Llama, BC, Alpaca<br>Netherlands; 2011; Llama, BC, Alpaca, Guanaco		DC serum: 155/65<br>DC Canary Island: 105/15<br>DC Oman: 50/50<br>Male: 50/0<br>Female: 105/50<br><2 years: 8/1<br>2-5 years: 9/0<br>>5 years: 88/64<br>Llama serum: 7/0<br>Alpaca serum: 24/0<br>BC serum: 4/0<br>Guanaco serum: 2/0		Quality: High
73	Reusken et al., 2014a[76]	Qatar; April 2014; DC	Nasal/Spring/>5 years/female: 12/5<br>Rectal: 12/3<br>Milk: 12/5	Serum/Spring/>5 years/female: 12/12<br>Milk: 12/12		Quality: Intermediate
74	Reusken et al., 2014b[77]	Ethiopia; 2010-2011; DC<br>Nigeria; 2010-2011; DC<br>Tunisia; 2009; DC		Serum: 750/617<br>Ethiopia: 188/181<br>Nigeria/Abattoir: 358/337<br>Tunisia: 204/99		Quality: High
75	Reusken et al., 2016[78]	Qatar; April 2015; DC, Alpaca	DC/Nasal/Spring: 10/0<br>DC/Female: 9/0<br>DC/Male: 1/0<br>Alpaca: 15/0	DC/Serum/Spring: 10/9<br>DC/Female: 9/8<br>DC/Male: 1/1<br>Alpaca: 15/15		Quality: High
76	Sabir et al., 2016[79]	Saudi Arabia; 2014-2015; DC	Nasal: 1309/159<br>Abattoir: 14/0<br>Farm: 133/14<br>Market: 1162/145<br>Local: 893/133<br>Imported: 407/26<br><2 years: 1014/128<br>2-5 years: 206/20			Quality: High
77	Salam et al., 2022[80]	Nigeria; 2016-2017; DC		Serum: 74/74<br>Female: 60/60<br>Male: 14/14		Quality: High
78	Saqib et al., 2017[81]	Pakistan; 2012-2015; DC		Serum: 565/315<br>Male: 217/96<br>Female: 348/127<br><2 years: 89/26<br>2-5 years: 208/62<br>>5 years: 268/135	• Seropositivity increases with age and is higher in males	Quality: High
79	Sayed et al., 2020[82]	Egypt; 2015-2016; DC		Serum/>5 years/Winter: 63/37<br>Female: 28/28<br>Male: 35/9<br>Imported: 33/32	• Seroprevalence is significantly associated with sampling locality, age and sex of animals and sampling locations	Quality: High
80	Shirato et al., 2015[83]	Japan; 2015; DC, BC	DC Nasal: 4/0<br>DC Oral: 9/0<br>DC Rectal: 18/0	BC serum: 5/0		Quality: High
81	Shirato et al., 2019[84]	Ethiopia; 2013; DC	Nasal: 258/39	Serum: 184/179	• Seroprevalence increases with age of the animals.	Quality: High
82	Sitawa et al., 2020[85]	Kenya; 2016-2017; DC		Serum: 1421/895<br>Free herd: 1292/891<br>Farm: 129/13<br><2 years: 380/167<br>2-5 years: 156/95<br>Female: 1129/752<br>Male: 292/153	• Seroprevalence also significantly differs due to animal age, sex, and production system.	Ranching and pastoralism animals were considered as farm and free herd, respectively<br>Quality: High
83	So et al., 2018[86]	Nigeria; 2015-2016; DC	Nasal: 2529/55<br><2 years: 38/4<br>Autumn: 1300/0<br>Winter: 1229/55			Quality: High

Ref	Author	Sampling country, time and animal	Molecular study results	Immunologic study results	Additional information	Remark
84	Thwiny et al., 2018[87]	Iraq; 2014-2015; DC		Plasma: 180/85<br><2 years: 44/39<br>2-5 years: 58/47<br>Male: 98/83<br>Female: 82/70	• There was no significant difference in seroprevalence between different camel breeds, age, and sex.	Quality: High
85	Tolah et al., 2020[88]	Saudi Arabia: 2016 to 2018; DC	Nasal: 1399/240<br>Imported: 1157/154<br>Local: 242/86<br><2 years: 451/120<br>Male: 800/194<br>Female: 364/46	Serum: 1399/1297<br>Imported: 1157/1085<br>Local: 242/212<br><2 years: 451/381<br>Male: 800/728<br>Female: 364/342	• Seroprevalence was increased with age.<br>• Viral RNA detection rate was higher in young than aged animals.<br>• RNA detection was higher in males.	Quality: High
86	Van Doremalen et el., 2017[89]	Jordan; May 2016; DC	Nasal: 39/27<br><2 years: 31/23<br>2-5 years: 8/4<br>Urogenital: 39/0<br>Rectal: 39/0	Serum: 39/27<br><2 years: 31/23<br>2-5 years: 8/4	• Viral shedding was detected only among animals below 3 years<br>• The seroprevalence increased with the age of camels	Quality: High
87	Wernery et al., 2015a[90]	UAE; March-June, 2014; DC	Nasal: 871/45<br><2 years: 209/11	Serum/Farm: 843/786<br><2 years: 108/92	• Aged animals had higher seroprevalence than young animals<br>• Young animals showed more viral RNA than aged animals	Quality: High
88	Wernery et al., 2015b[91]	UAE, 2015; DC		Serum: 254/234<br><2 years: 122/102	• MERS-CoV IgG seropositivity increases gradually with increase of camel calves' age	Quality: High
89	Woo et al., 2014[92]	UAE; 2013; DC	Rectal: 293/14	Serum: 59/58		Neutralization test was considered for analysis. Quality: High
90	Yusof et al., 2015[93]	UAE; 2014; DC	Nasal: 7803/126<br>Quarantine: 7570/101<br>Abattoir: 303/25<br>Public escorts: 30/0<br>Imported: 7470/101<br>Local: 333/25			Quality: High
91	Yusof et al., 2017[94]	UAE; 2015; DC	Nasal/Spring: 376/139<br>Local: 210/53<br>Imported: 106/53			Quality: High
92	Zhang et al., 2019[95]	Kenya; 2016-2018; DC		Serum: 891/584		Quality: High
93	Zhou et al., 2023[96]	Ethiopia; 2017-20; DC	Nasal: 1766/61<br>Turbinate: 484/10			Quality: Intermediate
94	Zohaib et al., 2018[97]	Pakistan; 2015-2018; DC	Nasal: 776/22	Serum: 1050/794<br>>5 years: 161/131<br>Female: 695/543<br>Male: 355/251<br>Farm: 183/125<br>Free herd:478/391	• Seroprevalence was significantly associated with age and sex of the animals and type of camel herd.<br>• Seroprevalence was not significantly different among different provinces of Pakistan.	Nomad and pastoralist animals were considered as free herd, whereas, sedentary camels were farmed. Quality: High

UAE: United Arab Emirates; BC: Bactrian camel; DC: Dromedary camel; HC: Hybrid camel

References:

1. M.M. Ababneh; Lafi, S.Q.; Abutarbush, S.M.; Khalifeh, M.S.; Hijazeen, Z.S.K.; Ramadneh, W.A.; Al Ameer, M.S.; Abukhalifeh, F.Y.; Kutkut, T.A.; Dodeen, R.A.; et al. Longitudinal and abattoir-based surveillance of MERS-CoV in camels in Jordan, 2018-2020. Heliyon **2021**, *7*, e08166, doi:10.1016/j.heliyon.2021.e08166.
2. Z.M. Al Hammadi; Chu, D.K.W.; Eltahir, Y.M.; Al Hosani, F.; Al Mulla, M.; Tarnini, W.; Hall, A.J.; Perera, R.a.P.M.; Abdelkhalek, M.M.; Peiris, J.S.M.; et al. Asymptomatic MERS-CoV infection in humans possibly linked to infected dromedaries imported from Oman to United Arab Emirates, May 2015. Emerging Infectious Diseases **2015**, *21*, 2197-2200, doi:10.3201/eid2112.151132.
3. S.F. Al Salihi; Alrodhan, M.A. Phylogenetic Analysis of MERSCoV in Human and Camels in Iraq. International Journal of Pharmaceutical Research and Allied Sciences **2017**, *6*, 120-129.
4. A.N. Alagaili; Briese, T.; Mishra, N.; Kapoor, V.; Sameroff, S.C.; De Wit, E.; Munster, V.J.; Hensley, L.E.; Zalmout, I.S.; Kapoor, A.; et al. Middle east respiratory syndrome coronavirus infection in dromedary camels in Saudi Arabia. mBio **2014**, *5*, doi:10.1128/mBio.00884-14.
5. A.N. Alagaili; Briese, T.; Mishra, N.; Kapoor, V.; Sameroff, S.C.; Burbelo, P.D.; De Wit, E.; Munster, V.J.; Hensley, L.E.; Zalmout, I.S.; et al. Erratum to Middle East respiratory syndrome coronavirus infection in dromedary camels in Saudi Arabia. mBio **2014**, *5*, doi:10.1128/mBio.01002-14.

6. S. Alexandersen; Kobinger, G.P.; Soule, G.; Wernery, U. Middle east respiratory syndrome coronavirus antibody reactors among camels in Dubai, United Arab Emirates, in 2005. Transboundary and Emerging Diseases **2014**, *61*, 105-108, doi:10.1111/tbed.12212.
7. M. Alfuwaires; Altaher, A.; Alhafufi, A.; Kandeel, M. Middle east respiratory syndrome coronavirus in healthy and diseased dromedaries. Journal of Camel Practice and Research **2017**, *24*, 217-220, doi:10.5958/2277-8934.2017.00036.4.
8. M.A. Ali; Shehata, M.M.; Gomaa, M.R.; Kandeil, A.; El-Shesheny, R.; Kayed, A.S.; El-Taweel, A.N.; Atea, M.; Hassan, N.; Bagato, O.; et al. Systematic, active surveillance for Middle East respiratory syndrome coronavirus in camels in Egypt. Emerging Microbes and Infections **2017**, *6*, doi:10.1038/emi.2016.130.
9. M.A. Ali; El-Shesheny, R.; Kandeil, A.; Shehata, M.; Elsokary, B.; Gomaa, M.; Hassan, N.; El Sayed, A.; El-Taweel, A.; Sobhy, H.; et al. Cross-sectional surveillance of middle east respiratory syndrome coronavirus (MERS-CoV) in dromedary camels and other mammals in Egypt, August 2015 to January 2016. Eurosurveillance **2017**, *22*, doi:10.2807/1560-7917.ES.2017.22.11.30487.
10. T.A. Aljasim; Almasoud, A.; Aljami, H.A.; Alenazi, M.W.; Alsagaby, S.A.; Alsaleh, A.N.; Alharbi, N.K. High rate of circulating MERS-CoV in dromedary camels at slaughterhouses in Riyadh, 2019. Viruses **2020**, *12*, doi:10.3390/v12111215.
11. A. Alshehri; Mir, N.A.; Miled, N. Detection of Middle East Respiratory Syndrome Coronavirus-Specific RNA and Anti-MERS-Receptor-Binding Domain Antibodies in Camel Milk from Different Regions of Saudi Arabia. Viral Immunol **2022**, *35*, 673-680, doi:10.1089/vim.2022.0045.
12. E.I. Azhar; El-Kafrawy, S.A.; Farraj, S.A.; Hassan, A.M.; Al-Saeed, M.S.; Hashem, A.M.; Madani, T.A. Evidence for camel-to-human transmission of MERS coronavirus. New England Journal of Medicine **2014**, *370*, 2499-2505, doi:10.1056/NEJMoa1401505.
13. D. Bold; Van Doremalen, N.; Myagmarsuren, O.; Zayat, B.; Munster, V.J.; Richt, J.A. Middle East Respiratory Syndrome-Coronavirus Seropositive Bactrian Camels, Mongolia. Vector-Borne and Zoonotic Diseases **2021**, *21*, 128-131.
14. S.M.S. Chan; Damdinjav, B.; Perera, R.a.P.M.; Chu, D.K.W.; Khishgee, B.; Enkhbold, B.; Poon, L.L.M.; Peiris, M. Absence of MERS-Coronavirus in bactrian camels, Southern Mongolia, November 2014. Emerging Infectious Diseases **2015**, *21*, 1269-1271, doi:10.3201/eid2107.150178.
15. D.K.W. Chu; Poon, L.L.M.; Gomaa, M.M.; Shehata, M.M.; Perera, R.a.P.M.; Zeid, D.A.; El Rifay, A.S.; Siu, L.Y.; Guan, Y.; Webby, R.J.; et al. MERS coronaviruses in dromedary camels, Egypt. Emerging Infectious Diseases **2014**, *20*, 1049-1053, doi:10.3201/eid2006.140299.
16. D.K. Chu; Oladipo, J.O.; Perera, R.A.; Kuranga, S.A.; Chan, S.M.; Poon, L.L.; Peiris, M. Middle east respiratory syndrome coronavirus (MERSCoV) in dromedary camels in nigeria, 2015. Eurosurveillance **2015**, *20*, 1-7, doi:10.2807/1560-7917.ES.2015.20.49.30086.
17. D.K.W. Chu; Hui, K.P.Y.; Perera, R.a.P.M.; Miguel, E.; Niemeyer, D.; Zhao, J.; Channappanavar, R.; Dudas, G.; Oladipo, J.O.; Traoré, A.; et al. MERS coronaviruses from camels in Africa exhibit region-dependent genetic diversity. Proceedings of the National Academy of Sciences of the United States of America **2018**, *115*, 3144-3149, doi:10.1073/pnas.1718769115.
18. V.M. Corman; Jores, J.; Meyer, B.; Younan, M.; Liljander, A.; Said, M.Y.; Gluecks, I.; Lattwein, E.; Bosch, B.J.; Drexler, J.F.; et al. Antibodies against MERS coronavirus in dromedary camels, Kenya, 1992-2013. Emerging Infectious Diseases **2014**, *20*, 1319-1322, doi:10.3201/eid2008.140596.
19. G. Crameri; Durr, P.A.; Barr, J.; Yu, M.; Graham, K.; Williams, O.J.; Kayali, G.; Smith, D.; Peiris, M.; Mackenzie, J.S.; et al. Absence of MERS-CoV antibodies in feral camels in Australia: Implications for the pathogen's origin and spread. One Health **2015**, *1*, 76-82, doi:10.1016/j.onehlt.2015.10.003.
20. D. David; Rotenberg, D.; Khinich, E.; Erster, O.; Bardenstein, S.; Van Straten, M.; Okba, N.M.A.; Raj, S.V.; Haagmans, B.L.; Miculitzki, M.; et al. Middle East respiratory syndrome coronavirus specific antibodies in naturally exposed Israeli llamas, alpacas and camels. One Health **2018**, *5*, 65-68, doi:10.1016/j.onehlt.2018.05.002.
21. S.L. Deem; Fèvre, E.M.; Kinnaird, M.; Browne, A.S.; Muloi, D.; Godeke, G.J.; Koopmans, M.; Reusken, C.B. Serological evidence of MERS-CoV antibodies in dromedary camels (camelus dromedaries) in laikipia county, Kenya. PLoS ONE **2015**, *10*, doi:10.1371/journal.pone.0140125.
22. S.L. Deem; Fèvre, E.M.; Kinnaird, M.; Browne, A.S.; Muloi, D.; Godeke, G.J.; Koopmans, M.; Reusken, C.B. Correction: Serological Evidence of MERS-CoV Antibodies in Dromedary Camels (Camelus dromedaries) in Laikipia County, Kenya. PLoS One **2017**, *12*, e0178310, doi:10.1371/journal.pone.0178310.
23. S. Eckstein; Ehmann, R.; Gritli, A.; Ben Yahia, H.; Diehl, M.; Wölfel, R.; Ben Rhaiem, M.; Stoecker, K.; Handrick, S.; Ben Moussa, M. Prevalence of middle east respiratory syndrome coronavirus in dromedary camels, tunisia. Emerging Infectious Diseases **2021**, *27*, 1964-1968, doi:10.3201/eid2707.204873.
24. A.A. Elfadil; Ahmed, A.G.; Abdalla, M.O.; Gumaa, E.; Osman, O.H.; Younis, A.E.; Al-Hafufi, A.N.; Saif, L.J.; Zaki, A.; Al-Rumaihi, A.; et al. Epidemiological study of Middle East respiratory syndrome coronavirus infection in dromedary camels in Saudi Arabia, April-May 2015. Revue scientifique et technique (International Office of Epizootics) **2018**, *37*, 985-997, doi:10.20506/rst.37.3.2901.
25. S.A. El-Kafrawy; Corman, V.M.; Tolah, A.M.; Al Masaudi, S.B.; Hassan, A.M.; Müller, M.A.; Bleicker, T.; Harakeh, S.M.; Alzahrani, A.A.; Alsaaidi, G.A.; et al. Enzootic patterns of Middle East respiratory syndrome coronavirus in imported African and local Arabian dromedary camels: a prospective genomic study. The Lancet Planetary Health **2019**, *3*, e521-e528, doi:10.1016/S2542-5196(19)30243-8.
26. D. Falzarano; Kamissoko, B.; De Wit, E.; Maïga, O.; Cronin, J.; Samaké, K.; Traoré, A.; Milne-Price, S.; Munster, V.J.; Sogoba, N.; et al. Dromedary camels in northern Mali have high seropositivity to MERS-CoV. One Health **2017**, *3*, 41-43, doi:10.1016/j.onehlt.2017.03.003.
27. E. Farag; Sikkema, R.S.; Mohamedani, A.A.; De Bruin, E.; Oude Munnink, B.B.; Chandler, F.; Kohl, R.; Van Der Linden, A.; Okba, N.M.A.; Haagmans, B.L.; et al. MERS-CoV in camels but not camel handlers, Sudan, 2015 and 2017. Emerging Infectious Diseases **2019**, *25*, 2333-2335, doi:10.3201/eid2512.190882.
28. M.S. Gaddafi; Faleke, O.O.; Yakubu, Y.; Garba, B.; Musawa, I.A.; Junaidu, A.U.; Magaji, A.A.; Alkali, B.R.; Aliyu, M.A. Seroprevalence of themiddle east respiratory syndrome coronavirus antibodies in camels from two international animal control-posts, Kebbi State, Nigeria. Journal of Animal Health and Production **2020**, *8*, 50-54, doi:10.14737/journal.jahp/2020/8.2.50.54.
29. E.G. Gardner; Kiambi, S.; Sitawa, R.; Kelton, D.; Kimutai, J.; Poljak, Z.; Tadesse, Z.; Von Dobschuetz, S.; Wiersma, L.; Greer, A.L. Force of infection of Middle East respiratory syndrome in dromedary camels in Kenya. Epidemiology and infection **2019**, *147*, e275, doi:10.1017/S0950268819001663.
30. C. Gutiérrez; Tejedor-Junco, M.T.; González, M.; Lattwein, E.; Renneker, S. Presence of antibodies but no evidence for circulation of MERS-CoV in dromedaries on the Canary Islands, 2015. Eurosurveillance **2015**, *20*, doi:10.2807/1560-7917.ES.2015.20.37.30019.

31. B.L. Haagmans; Al Dhahiry, S.H.S.; Reusken, C.B.E.M.; Raj, V.S.; Galiano, M.; Myers, R.; Godeke, G.J.; Jonges, M.; Farag, E.; Diab, A.; et al. Middle East respiratory syndrome coronavirus in dromedary camels: An outbreak investigation. The Lancet Infectious Diseases **2014**, *14*, 140-145, doi:10.1016/S1473-3099(13)70690-X.
32. J.L. Harcourt; Rudoler, N.; Tamin, A.; Leshem, E.; Rasis, M.; Giladi, M.; Haynes, L.M. The prevalence of Middle East respiratory syndrome coronavirus (MERS-CoV) antibodies in dromedary camels in Israel. Zoonoses and Public Health **2018**, *65*, 749-754, doi:10.1111/zph.12482.
33. R. Harrath; Abu Duhier, F.M. Sero-prevalence of Middle East respiratory syndrome coronavirus (MERS-CoV) specific antibodies in dromedary camels in Tabuk, Saudi Arabia. Journal of Medical Virology **2018**, *90*, 1285-1289, doi:10.1002/jmv.25186.
34. A.S. Hasan; Alı, K.S.; Saleh, M.K. Cross-sectional study of Middle East respiratory syndrome coronavirus in humans and dromedary camels in Diyala, Iraq. Turk J Med Sci **2022**, *52*, 910-916, doi:10.55730/1300-0144.5390.
35. M.G. Hemida; Perera, R.A.; Wang, P.; Alhammadi, M.A.; Siu, L.Y.; Li, M.; Poon, L.L.; Saif, L.; Alnaeem, A.; Peiris, M. Middle east respiratory syndrome (MERS) coronavirus seroprevalence in domestic livestock in Saudi Arabia, 2010 to 2013. Eurosurveillance **2013**, *18*, doi:10.2807/1560-7917.ES2013.18.50.20659.
36. M.G. Hemida; Perera, R.A.; Al Jassim, R.A.; Kayali, G.; Siu, L.Y.; Wang, P.; Chu, K.W.; Perlman, S.; Ali, M.A.; Alnaeem, A.; et al. Seroepidemiology of middle east respiratory syndrome (MERS) coronavirus in Saudi Arabia (1993) and Australia (2014) and characterisation of assay specificity. Eurosurveillance **2014**, *19*, doi:10.2807/1560-7917.ES2014.19.23.20828.
37. M.G. Hemida; Chu, D.K.W.; Poon, L.L.M.; Perera, R.a.P.M.; Alhammadi, M.A.; Ng, H.Y.; Siu, L.Y.; Guan, Y.; Alnaeem, A.; Peiris, M. Mers coronavirus in dromedary camel herd, Saudi Arabia. Emerging Infectious Diseases **2014**, *20*, 1231-1234, doi:10.3201/eid2007.140571.
38. M.G. Hemida; Alnaeem, A.; Chu, D.K.W.; Perera, R.a.P.M.; Chan, S.M.S.; Almathen, F.; Yau, E.; Ng, B.C.Y.; Webby, R.J.; Poon, L.L.M.; et al. Longitudinal study of Middle East Respiratory Syndrome coronavirus infection in dromedary camel herds in Saudi Arabia, 2014–2015. Emerging Microbes and Infections **2017**, *6*, 1-7, doi:10.1038/emi.2017.44.
39. M.G. Hemida; Waheed, M.; Ali, A.M.; Alnaeem, A. Detection of the Middle East respiratory syndrome coronavirus in dromedary camel's seminal plasma in Saudi Arabia 2015–2017. Transboundary and Emerging Diseases **2020**, *67*, 2609-2614, doi:10.1111/tbed.13610.
40. M.G. Hemida; Al, A.M.; Alhammadi, M.; Alnaeem, A. The Middle East respiratory syndrome coronavirus in the breath of some infected dromedary camels (Camelus dromedarius). Epidemiology and Infection **2020**, doi:10.1017/S0950268820002459.
41. M.G. Hemida; Chu, D.K.W.; Chor, Y.Y.; Cheng, S.M.S.; Poon, L.L.M.; Alnaeem, A.; Peiris, M. Phylogenetic analysis of MERS-CoV in a camel abattoir, Saudi Arabia, 2016-2018. Emerging Infectious Diseases **2020**, *26*, 3089-3091, doi:10.3368/CL.60.4.549.
42. M.G. Hemida; Ali, A.M.; Alnaeem, A. The Middle East respiratory syndrome coronavirus (MERS-CoV) nucleic acids detected in the saliva and conjunctiva of some naturally infected dromedary camels in Saudi Arabia -2019. Zoonoses and Public Health **2021**, *68*, 353-357, doi:10.1111/zph.12816.
43. P. Holloway; Gibson, M.; Van Doremalen, N.; Nash, S.; Holloway, T.; Letko, M.; Cardwell, J.M.; Al Omari, B.; Al-Majali, A.; Abu-Basha, E.; et al. Risk Factors for Middle East Respiratory Syndrome Coronavirus Infection among Camel Populations, Southern Jordan, 2014-2018. Emerg Infect Dis **2021**, *27*, 2301-2311, doi:10.3201/eid2709.203508.
44. A. Islam; Epstein, J.H.; Rostal, M.K.; Islam, S.; Rahman, M.Z.; Hossain, M.E.; Uzzaman, M.S.; Munster, V.J.; Peiris, M.; Flora, M.S.; et al. Middle east respiratory syndrome Coronavirus antibodies in dromedary camels, Bangladesh, 2015. Emerging Infectious Diseases **2018**, *24*, 926-928, doi:10.3201/eid2405.171192.
45. A. Kandeil; Gomaa, M.; Nageh, A.; Shehata, M.M.; Kayed, A.E.; Sabir, J.S.M.; Abiadh, A.; Jrijer, J.; Amr, Z.; Said, M.A.; et al. Middle east respiratory syndrome coronavirus (Mers-cov) in dromedary camels in africa and middle east. Viruses **2019**, *11*, doi:10.3390/v11080717.
46. K. Karamendin; Seidalina, A.; Sabyrzhan, T.; Nuralibekov, S.; Kasymbekov, Y.; Suleimenova, S.; Khan, E.; Alikhanov, O.; Narsha, U.; Erkekulova, K.; et al. Serological Screening for Middle East Respiratory Syndrome Coronavirus and Hepatitis E Virus in Camels in Kazakhstan. Pathogens **2022**, *11*, doi:10.3390/pathogens11111224.
47. S. Kasem; Qasim, I.; Al-Doweriej, A.; Hashim, O.; Alkarar, A.; Abu-Obeida, A.; Saleh, M.; Al-Hofufi, A.; Al-Ghadier, H.; Hussien, R.; et al. The prevalence of Middle East respiratory Syndrome coronavirus (MERS-CoV) infection in livestock and temporal relation to locations and seasons. Journal of Infection and Public Health **2018**, *11*, 884-888, doi:10.1016/j.jiph.2018.01.004.
48. S. Kasem; Qasim, I.; Al-Hufofi, A.; Hashim, O.; Alkarar, A.; Abu-Obeida, A.; Gaafer, A.; Elfadil, A.; Zaki, A.; Al-Romaihi, A.; et al. Cross-sectional study of MERS-CoV-specific RNA and antibodies in animals that have had contact with MERS patients in Saudi Arabia. Journal of Infection and Public Health **2018**, *11*, 331-338, doi:10.1016/j.jiph.2017.09.022.
49. A.I. Khalafalla; Lu, X.; Al-Mubarak, A.I.A.; Dalab, A.H.S.; Al-Busadah, K.a.S.; Erdman, D.D. MERS-CoV in upper respiratory tract and lungs of dromedary camels, Saudi Arabia, 2013–2014. Emerging Infectious Diseases **2015**, *21*, 1153-1158, doi:10.3201/eid2107.150070.
50. S. Kiambi; Corman, V.M.; Sitawa, R.; Githinji, J.; Ngoci, J.; Ozomata, A.S.; Gardner, E.; Von Dobschuetz, S.; Morzaria, S.; Kimutai, J.; et al. Detection of distinct MERS-Coronavirus strains in dromedary camels from Kenya, 2017. Emerging Microbes and Infections **2018**, *7*, doi:10.1038/s41426-018-0193-z.
51. H.J. Kim; Choi, J.S.; Nam, H.M.; Kang, H.E. Absence of MERS-CoV in domestic camels, Republic of Korea, 2015. International Journal of Infectious Diseases **2016**, *53*, 127-127, doi:10.1016/j.ijid.2016.11.315.
52. S. Lado; Elbers, J.P.; Plasil, M.; Loney, T.; Weidinger, P.; Camp, J.V.; Kolodziejek, J.; Futas, J.; Kannan, D.A.; Orozco-Terwengel, P.; et al. Innate and adaptive immune genes associated with mers-cov infection in dromedaries. Cells **2021**, *10*, doi:10.3390/cells10061291.
53. S.K.P. Lau; Li, K.S.M.; Luk, H.K.H.; He, Z.; Teng, J.L.L.; Yuen, K.Y.; Wernery, U.; Woo, P.C.Y. Middle east respiratory syndrome coronavirus antibodies in bactrian and hybrid camels from Dubai. mSphere **2020**, *5*, doi:10.1128/mSphere.0898-19.
54. Y. Li; Khalafalla, A.I.; Paden, C.R.; Yusof, M.F.; Eltahir, Y.M.; Al Hammadi, Z.M.; Tao, Y.; Queen, K.; Al Hosani, F.; Gerber, S.I.; et al. Identification of diverse viruses in upper respiratory samples in dromedary camels from United Arab Emirates. PLoS ONE **2017**, *12*, doi:10.1371/journal.pone.0184718.
55. R. Liu; Wen, Z.; Wang, J.; Ge, J.; Chen, H.; Bu, Z. Absence of Middle East respiratory syndrome coronavirus in Bactrian camels in the West Inner Mongolia Autonomous Region of China: Surveillance study results from July 2015. Emerging Microbes and Infections **2015**, *4*, doi:10.1038/emi.2015.73.
56. Z.A. Memish; Cotten, M.; Meyer, B.; Watson, S.J.; Alsahafi, A.J.; Al Rabeeah, A.A.; Corman, V.M.; Sieberg, A.; Makhdoom, H.Q.; Assiri, A.; et al. Human Infection with MERS coronavirus after exposure to infected camels, Saudi Arabia, 2013. Emerging Infectious Diseases **2014**, *20*, 1012-1015, doi:10.3201/eid2006.140402.

57. B. Meyer; Müller, M.A.; Corman, V.M.; Reusken, C.B.E.M.; Ritz, D.; Godeke, G.J.; Lattwein, E.; Kallies, S.; Siemens, A.; Van Beek, J.; et al. Antibodies against MERS coronavirus in dromedaries, United Arab Emirates, 2003 and 2013. Emerging Infectious Diseases **2014**, *20*, 552-559, doi:10.3201/eid2004.131746.
58. B. Meyer; Juhasz, J.; Barua, R.; Das Gupta, A.; Hakimuddin, F.; Corman, V.M.; Müller, M.A.; Wernery, U.; Drosten, C.; Nagy, P. Time course of MERS-CoV infection and immunity in dromedary camels. Emerging Infectious Diseases **2016**, *22*, 2171-2173, doi:10.3201/eid2212.160382.
59. E. Miguel; Perera, R.a.P.M.; Baubekova, A.; Chevalier, V.; Faye, B.; Akhmetsadykov, N.; Ng, C.Y.; Roger, F.; Peiris, M. Absence of Middle East respiratory syndrome coronavirus in camelids, Kazakhstan, 2015. Emerging Infectious Diseases **2016**, *22*, 555-557, doi:10.3201/eid2203.151284.
60. E. Miguel; Chevalier, V.; Ayelet, G.; Ben Bencheikh, M.N.; Boussini, H.; Chu, D.K.; El Berbri, I.; Fassi-Fihri, O.; Faye, B.; Fekadu, G.; et al. Risk factors for MERS coronavirus infection in dromedary camels in Burkina Faso, Ethiopia, and Morocco, 2015. Eurosurveillance **2017**, *22*, doi:10.2807/1560-7917.ES.2017.22.13.30498.
61. L. Moazemi-Goudarzi; Ziafatikafi, Z.; Seyedasgari, F.; Najafi, H.; Hashemzadeh, M.; Aghaeean, L.; Ghalyanchilangeroudi, A. Molecular Detection of Middle East Respiratory Syndrome Coronavirus from Dromedary Camels Illegally Transferred to Iran. Acta Veterinaria Eurasia **2022**, *48*, 117-122, doi:10.54614/actavet.2022.21076.
62. K.A. Mohran; Farag, E.A.; Reusken, C.B.; Raj, V.S.; Lamers, M.M.; Pas, S.D.; Voermans, J.; Smits, S.L.; Alhajri, M.M.; Alhajri, F.; et al. The sample of choice for detecting Middle East respiratory syndrome coronavirus in asymptomatic dromedary camels using real-time reversetranscription polymerase chain reaction. Rev Sci Tech **2016**, *35*, 905-911, doi:10.20506/rst.35.3.2578.
63. S.A. Muhairi; Hosani, F.A.; Eltahir, Y.M.; Mulla, M.A.; Yusof, M.F.; Serhan, W.S.; Hashem, F.M.; Elsayed, E.A.; Marzoug, B.A.; Abdelazim, A.S. Epidemiological investigation of Middle East respiratory syndrome coronavirus in dromedary camel farms linked with human infection in Abu Dhabi Emirate, United Arab Emirates. Virus Genes **2016**, *52*, 848-854, doi:10.1007/s11262-016-1367-1.
64. M.A. Müller; Corman, V.M.; Jores, J.; Meyer, B.; Younan, M.; Liljander, A.; Bosch, B.J.; Lattwein, E.; Hilali, M.; Musa, B.E.; et al. Mers coronavirus neutralizing antibodies in camels, eastern Africa, 1983–1997. Emerging Infectious Diseases **2014**, *20*, 2093-2095, doi:10.3201/eid2012.141026.
65. P. Munyua; Corman, V.M.; Bitek, A.; Osoro, E.; Meyer, B.; Müller, M.A.; Lattwein, E.; Thumbi, S.M.; Murithi, R.; Widdowson, M.A.; et al. No serologic evidence of middle east respiratory syndrome coronavirus infection among camel farmers exposed to highly seropositive camel herds: A household linked study, Kenya, 2013. American Journal of Tropical Medicine and Hygiene **2017**, *96*, 1318-1324, doi:10.4269/ajtmh.16-0880.
66. I. Ngere; Munyua, P.; Harcourt, J.; Hunsperger, E.; Thornburg, N.; Muturi, M.; Osoro, E.; Gachohi, J.; Bodha, B.; Okotu, B.; et al. High MERS-CoV seropositivity associated with camel herd profile, husbandry practices and household socio-demographic characteristics in Northern Kenya. Epidemiology and Infection **2020**, doi:10.1017/S0950268820002939.
67. I. Ngere; Hunsperger, E.A.; Tong, S.; Oyugi, J.; Jaoko, W.; Harcourt, J.L.; Thornburg, N.J.; Oyas, H.; Muturi, M.; Osoro, E.M.; et al. Outbreak of Middle East Respiratory Syndrome Coronavirus in Camels and Probable Spillover Infection to Humans in Kenya. Viruses **2022**, *14*, doi:10.3390/v14081743.
68. N. Nowotny; Kolodziejek, J. Middle East respiratory syndrome coronavirus (MERS-CoV) in dromedary camels, Oman, 2013. Euro Surveill **2014**, *19*, 20781, doi:10.2807/1560-7917.es2014.19.16.20781.
69. S. Ommeh; Zhang, W.; Zohaib, A.; Chen, J.; Zhang, H.J.; Hu, B.; Ge, X.Y.; Yang, X.L.; Masika, M.; Obanda, V.; et al. Genetic Evidence of Middle East Respiratory Syndrome Coronavirus (MERS-Cov) and Widespread Seroprevalence among Camels in Kenya (vol 33, pg 484, 2018). Virologica Sinica **2019**, *34*, 115-115, doi:10.1007/s12250-019-00092-6.
70. S. Ommeh; Zhang, W.; Zohaib, A.; Chen, J.; Zhang, H.; Hu, B.; Ge, X.Y.; Yang, X.L.; Masika, M.; Obanda, V.; et al. Correction to: Genetic Evidence of Middle East Respiratory Syndrome Coronavirus (MERS-Cov) and Widespread Seroprevalence among Camels in Kenya. Virol Sin **2019**, *34*, 115, doi:10.1007/s12250-019-00092-6.
71. M.B. Orynbayev; Hitch, A.T.; Kerimbayev, A.A.; Nissanova, R.K.; Sultankulova, K.T.; Rystayeva, R.A.; Omarova, Z.D.; Kassenov, M.M.; Tailakova, E.T.; Smith, G.J.D.; et al. Serological exposure in Bactrian and dromedary camels in Kazakhstan to a MERS or MERS-like coronavirus. Transbound Emerg Dis **2022**.
72. R.A. Perera; Wang, P.; Gomaa, M.R.; El-Shesheny, R.; Kandeil, A.; Bagato, O.; Siu, L.Y.; Shehata, M.M.; Kayed, A.S.; Moatasim, Y.; et al. Seroepidemiology for MERS coronavirus using microneutralisation and pseudoparticle virus neutralisation assays reveal a high prevalence of antibody in dromedary camels in Egypt, june 2013. Eurosurveillance **2013**, *18*, doi:10.2807/1560-7917.ES2013.18.36.20574.
73. V.S. Raj; Farag, E.a.B.A.; Reusken, C.B.E.M.; Lamers, M.M.; Pas, S.D.; Voermans, J.; Smits, S.L.; Osterhaus, A.D.M.E.; Al-Mawlawi, N.; Al-Romaihi, H.E.; et al. Isolation of MERS coronavirus from dromedary camel, Qatar, 2014. Emerging Infectious Diseases **2014**, *20*, 1339-1342, doi:10.3201/eid2008.140663.
74. C.B. Reusken; Ababneh, M.; Raj, V.S.; Meyer, B.; Eljarah, A.; Abutarbush, S.; Godeke, G.J.; Bestebroer, T.M.; Zutt, I.; Müller, M.A.; et al. Middle east respiratory syndrome coronavirus (MERS-CoV) serology in major livestock species in an affected region in Jordan, june to September 2013. Eurosurveillance **2013**, *18*, doi:10.2807/1560-7917.ES2013.18.50.20662.
75. C.B.E.M. Reusken; Haagmans, B.L.; Müller, M.A.; Gutierrez, C.; Godeke, G.J.; Meyer, B.; Muth, D.; Raj, V.S.; Vries, L.S.D.; Corman, V.M.; et al. Middle East respiratory syndrome coronavirus neutralising serum antibodies in dromedary camels: A comparative serological study. The Lancet Infectious Diseases **2013**, *13*, 859-866, doi:10.1016/S1473-3099(13)70164-6.
76. C.B. Reusken; Farag, E.A.; Jonges, M.; Godeke, G.J.; El-Sayed, A.M.; Pas, S.D.; Raj, V.S.; Mohran, K.A.; Moussa, H.A.; Ghobashy, H.; et al. Middle east respiratory syndrome coronavirus (MERS-CoV) RNA and neutralising antibodies in milk collected according to local customs from dromedary camels, Qatar, April 2014. Eurosurveillance **2014**, *19*, doi:10.2807/1560-7917.ES2014.19.23.20829.
77. C.B.E.M. Reusken; Messadi, L.; Feyisa, A.; Ularamu, H.; Godeke, G.J.; Danmarwa, A.; Dawo, F.; Jemli, M.; Melaku, S.; Shamaki, D.; et al. Geographic distribution of MERS coronavirus among dromedary camels, Africa. Emerging Infectious Diseases **2014**, *20*, 1370-1374, doi:10.3201/eid2008.140590.
78. C.B.E.M. Reusken; Schilp, C.; Raj, V.S.; De Bruin, E.; Kohl, R.H.G.; Farag, E.a.B.A.; Haagmans, B.L.; Al-Romaihi, H.; Le Grange, F.; Bosch, B.J.; et al. MERS-CoV infection of alpaca in a region whereMERS-CoV is endemic. Emerging Infectious Diseases **2016**, *22*, 1129-1131, doi:10.3201/eid2206.152113.
79. J.S.M. Sabir; Lam, T.T.Y.; Ahmed, M.M.M.; Li, L.; Shen, Y.; Abo-Aba, S.E.M.; Qureshi, M.I.; Abu-Zeid, M.; Zhang, Y.; Khiyami, M.A.; et al. Co-circulation of three camel coronavirus species and recombination of MERS-CoVs in Saudi Arabia. Science **2016**, *351*, 81-84, doi:10.1126/science.aac8608.
80. S.P. Salam; Sabo Nok Kia, G.; Oladayo, F.O.; Ugochukwu, I.C.I. Serosurvey for Middle East respiratory syndrome coronavirus antibody in dromedary camels and human patients at a secondary care hospital, Illela, Northwest Nigeria. Comp Clin Path **2022**, 1-10.

81. M. Saqib; Sieberg, A.; Hussain, M.H.; Mansoor, M.K.; Zohaib, A.; Lattwein, E.; Müller, M.A.; Drosten, C.; Corman, V.M. Serologic evidence for MERS-CoV infection in Dromedary Camels, Punjab, Pakistan, 2012–2015. Emerging Infectious Diseases **2017**, *23*, 550-551, doi:10.3201/eid2303.161285.
82. A.S.M. Sayed; Malek, S.S.; Abushahba, M.F.N. Seroprevalence of Middle East Respiratory Syndrome Corona Virus in dromedaries and their traders in upper Egypt. Journal of Infection in Developing Countries **2020**, *14*, 191-198, doi:10.3855/jidc.10862.
83. K. Shirato; Azumano, A.; Nakao, T.; Hagihara, D.; Ishida, M.; Tamai, K.; Yamazaki, K.; Kawase, M.; Okamoto, Y.; Kawakami, S.; et al. Middle east respiratory syndrome coronavirus infection not found in camels in Japan. Japanese Journal of Infectious Diseases **2015**, *68*, 256-258, doi:10.7883/yoken.JJID.2015.094.
84. K. Shirato; Melaku, S.K.; Kawachi, K.; Nao, N.; Iwata-Yoshikawa, N.; Kawase, M.; Kamitani, W.; Matsuyama, S.; Tessema, T.S.; Sentsui, H. Middle east respiratory syndrome coronavirus in dromedaries in Ethiopia is antigenically different from the Middle East isolate EMC. Frontiers in Microbiology **2019**, *10*, doi:10.3389/fmicb.2019.01326.
85. R. Sitawa; Folorunso, F.; Obonyo, M.; Apamaku, M.; Kiambi, S.; Gikonyo, S.; Kiptiness, J.; Njagi, O.; Githinji, J.; Ngoci, J.; et al. Risk factors for serological evidence of MERS-CoV in camels, Kenya, 2016–2017. Preventive Veterinary Medicine **2020**, *185*, doi:10.1016/j.prevetmed.2020.105197.
86. T.Y. So Ray; Perera Ranawaka, A.P.M.; Oladipo Jamiu, O.; Chu Daniel, K.W.; Kuranga Sulyman, A.; Kin-Ho, C.; Lau Eric, H.Y.; Cheng Samuel, M.S.; Poon Leo, L.M.; Webby Richard, J.; et al. Lack of serological evidence of middle east respiratory syndrome coronavirus infection in virus exposed camel abattoir workers in Nigeria, 2016. Eurosurveillance **2018**, *23*, doi:10.2807/1560-7917.ES.2018.23.32.1800175.
87. H.T. Thwiny; Al Hamed, T.A.; Nazzal, A.R. Seroepidemiological study of Middle East respiratory syndrome (MERS) virus infection in Iraqi dromedary camels. Veterinarski Arhiv **2018**, *88*, 191-200, doi:10.24099/vet.arhiv.161224.
88. A.M. Tolah; Al Masaudi, S.B.; El-Kafrawy, S.A.; Mirza, A.A.; Harakeh, S.M.; Hassan, A.M.; Alsaadi, M.A.; Alzahrani, A.A.; Alsaaidi, G.A.; Amor, N.M.S.; et al. Cross-sectional prevalence study of MERS-CoV in local and imported dromedary camels in Saudi Arabia, 2016-2018. Plos One **2020**, *15*, doi:10.1371/journal.pone.0232790.
89. N. Van Doremalen; Hijazeen, Z.S.K.; Holloway, P.; Al Omari, B.; Mcdowell, C.; Adney, D.; Talafha, H.A.; Guitian, J.; Steel, J.; Amarin, N.; et al. High prevalence of middle east respiratory coronavirus in young dromedary camels in Jordan. Vector-Borne and Zoonotic Diseases **2017**, *17*, 155-159, doi:10.1089/vbz.2016.2062.
90. U. Wernery; Corman, V.M.; Wong, E.Y.M.; Tsang, A.K.L.; Muth, D.; Lau, S.K.P.; Khazanehdari, K.; Zirkel, F.; Ali, M.; Nagy, P.; et al. Acute middle east respiratory syndrome coronavirus infection in livestock dromedaries, Dubai, 2014. Emerging Infectious Diseases **2015**, *21*, 1019-1022, doi:10.3201/eid2106.150038.
91. U. Wernery; El Rasoul, I.; Wong, E.Y.M.; Joseph, M.; Chen, Y.; Jose, S.; Tsang, A.K.L.; Patteril, N.a.G.; Chen, H.; Elizabeth, S.K.; et al. A phylogenetically distinct Middle East respiratory syndrome coronavirus detected in a dromedary calf from a closed dairy herd in Dubai with rising seroprevalence with age. Emerging Microbes and Infections **2015**, *4*, doi:10.1038/emi.2015.74.
92. P.C. Woo; Lau, S.K.; Wernery, U.; Wong, E.Y.; Tsang, A.K.; Johnson, B.; Yip, C.C.; Lau, C.C.; Sivakumar, S.; Cai, J.P.; et al. Novel betacoronavirus in dromedaries of the Middle East, 2013. Emerg Infect Dis **2014**, *20*, 560-572, doi:10.3201/eid2004.131769.
93. M.F. Yusof; Eltahir, Y.M.; Serhan, W.S.; Hashem, F.M.; Elsayed, E.A.; Marzoug, B.A.; Abdelazim, A.S.; Bensalah, O.K.A.; Al Muhairi, S.S. Prevalence of Middle East respiratory syndrome coronavirus (MERS-CoV) in dromedary camels in Abu Dhabi Emirate, United Arab Emirates. Virus Genes **2015**, *50*, 509-513, doi:10.1007/s11262-015-1174-0.
94. M.F. Yusof; Queen, K.; Eltahir, Y.M.; Paden, C.R.; Al Hammadi, Z.M.a.H.; Tao, Y.; Li, Y.; Khalafalla, A.I.; Shi, M.; Zhang, J.; et al. Diversity of Middle East respiratory syndrome coronaviruses in 109 dromedary camels based on full-genome sequencing, Abu Dhabi, United Arab Emirates. Emerging Microbes and Infections **2017**, *6*, 1-10, doi:10.1038/emi.2017.89.
95. W. Zhang; Zheng, X.S.; Agwanda, B.; Ommeh, S.; Zhao, K.; Lichoti, J.; Wang, N.; Chen, J.; Li, B.; Yang, X.L.; et al. Serological evidence of MERS-CoV and HKU8-related CoV co-infection in Kenyan camels. Emerging Microbes and Infections **2019**, *8*, 1528-1534, doi:10.1080/22221751.2019.1679610.
96. Z. Zhou; Ali, A.; Walelign, E.; Demissie, G.F.; El Masry, I.; Abayneh, T.; Getachew, B.; Krishnan, P.; Ng, D.Y.M.; Gardner, E.; et al. Genetic diversity and molecular epidemiology of Middle East Respiratory Syndrome Coronavirus in dromedaries in Ethiopia, 2017-2020. Emerg Microbes Infect **2023**, *12*, e2164218, doi:10.1080/22221751.2022.2164218.
97. A. Zohaib; Saqib, M.; Athar, M.A.; Chen, J.; Sial, A.U.R.; Khan, S.; Taj, Z.; Sadia, H.; Tahir, U.; Tayyab, M.H.; et al. Countrywide Survey for MERS-Coronavirus Antibodies in Dromedaries and Humans in Pakistan. Virologica Sinica **2018**, *33*, 410-417, doi:10.1007/s12250-018-0051-0.
