# Supplementary material for: Global patterns of Middle East respiratory syndrome coronavirus (MERS-CoV) prevalence and seroprevalence in camels: A systematic review and meta-analysis
Source: One Health. 2023 May 8;16:100561. doi: 10.1016/j.onehlt.2023.100561 (PMC10166617; doi:10.1016/j.onehlt.2023.100561)
Supplement: Supplementary file 4 — Supplementary material 4 [file mmc4.pdf]

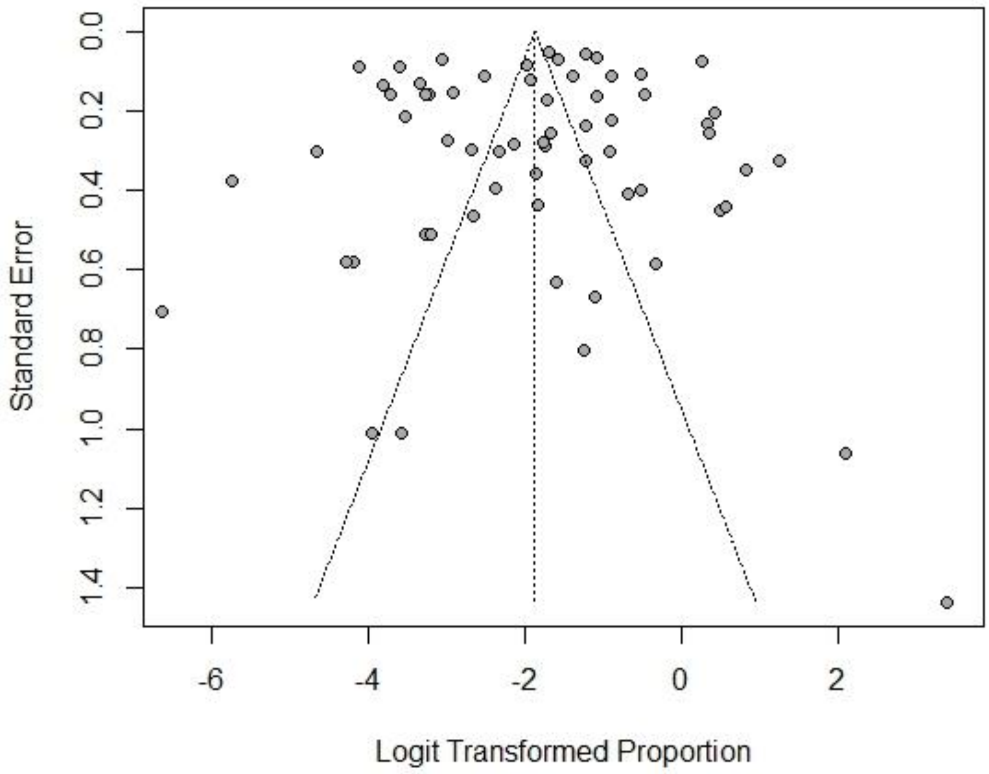

Supplementary Fig. 1. Funnel plot for examination of publication bias of the seroprevalence of MERS-CoV in dromedary camel.

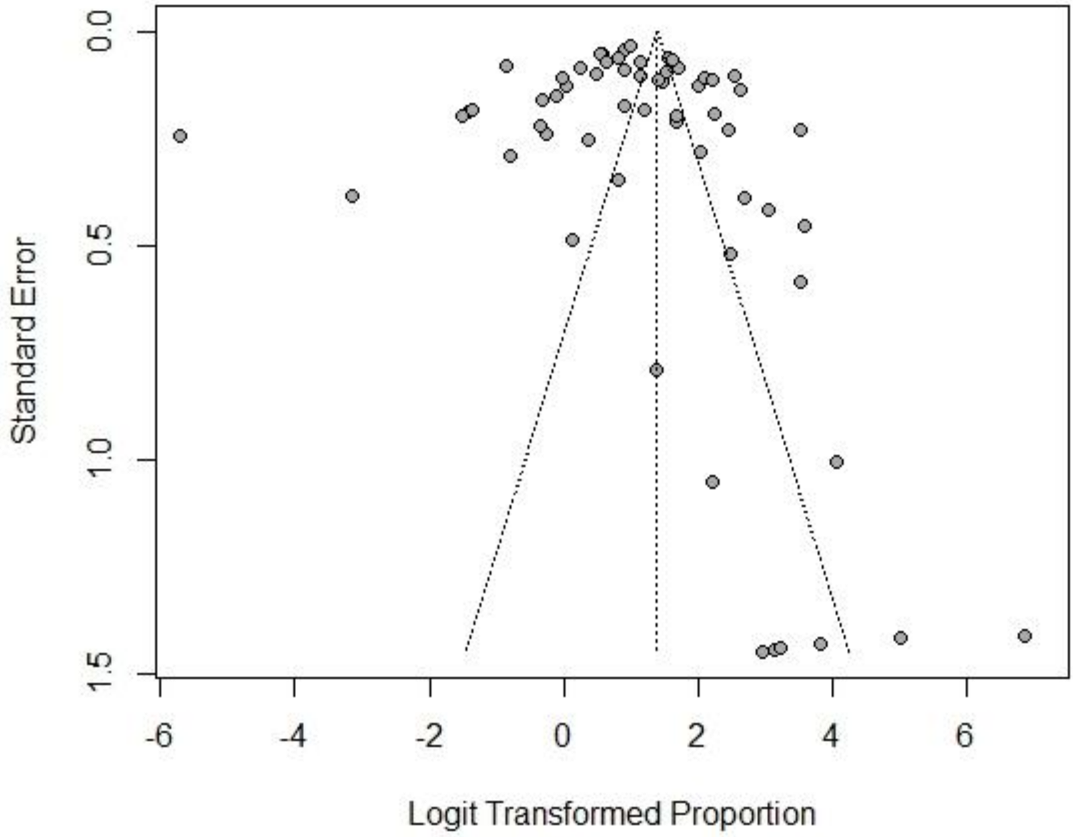

Supplementary Fig. 2. Funnel plot for examination of publication bias of the of MERS-CoV RNA prevalence in dromedary camel.
